# CDK4/6 inhibitors sensitize gammaherpesvirus-infected tumor cells to T-cell killing by enhancing expression of immune surface molecules

**DOI:** 10.1186/s12967-022-03400-z

**Published:** 2022-05-13

**Authors:** Yiquan Wu, Prabha Shrestha, Natalie M. Heape, Robert Yarchoan

**Affiliations:** HIV and AIDS Malignancy Branch, Center for Cancer Research, National Cancer Institute, Building 10, Rm. 6N106, MSC 1868, 10 Center Drive, Bethesda, MD 20892-1868 USA

**Keywords:** Gammaherpesvirus, KSHV, Kaposi sarcoma, EBV, MHC-I, Viral malignancy, Lymphoma, Immune evasion, CDK inhibitor, Cyclin

## Abstract

**Background:**

The two oncogenic human gammaherpesviruses, Kaposi sarcoma-associated herpesvirus (KSHV) and Epstein–Barr virus (EBV), both downregulate immune surface molecules, such as MHC-I, ICAM-1, and B7-2, enabling them to evade T-cell and natural killer cell immunity. Both also either encode for human cyclin homologues or promote cellular cyclin activity, and this has been shown to be important for proliferation and survival of gammaherpesvirus-induced tumors. CDK4/6 inhibitors, which are approved for certain breast cancers, have been shown to enhance expression of MHC-I in cell lines and murine models of breast cancer, and this was attributed to activation of interferons by endogenous retrovirus elements. However, it was not known if this would occur in gammaherpesvirus-induced tumors in which interferons are already activated.

**Methods:**

Multiple KSHV/EBV-infected cell lines were treated with CDK4/6 inhibitors. The growth of viable cells and expression of surface markers was assessed. T cell activation stimulated by the treated cells was assayed by a T-cell activation bioassay. Both viral and host gene expression was surveyed using RT-qPCR.

**Results:**

Three CDK4/6 inhibitors, abemaciclib, palbociclib, and ribociclib, inhibited cell growth in KSHV-induced primary effusion lymphoma (PEL) and EBV positive Burkitt’s lymphoma (BL) cell lines, and KSHV-infected human umbilical vein endothelial cells (HUVECs). Moreover, CDK4/6 inhibitors increased mRNA and surface expression of MHC-I in all three and prevented downregulation of MHC-I surface expression during lytic replication in KSHV-infected cells. CDK4/6 inhibitors also variably increased mRNA and surface expression of ICAM-1 and B7-2 in the tested lines. Abemaciclib also significantly enhanced T-cell activation induced by treated PEL and BL cells. Certain gammaherpesvirus genes as well as endogenous retrovirus (ERV) 3–1 genes were enhanced by CDK4/6 inhibitors in most PEL and BL lines and this enhancement was associated with expression of gamma interferon-induced genes including MHC-I.

**Conclusions:**

These observations provide evidence that CDK4/6 inhibitors can induce expression of surface immune markers MHC-I, B7-2, and ICAM-1 in gammaherpesvirus-infected cell lines and induce virus-specific immunity. They can thus thwart virus-induced immune evasion. These effects, along with their direct effects on KSHV- or EBV-induced tumors, provide a rational for the clinical testing of these drugs in these tumors.

**Supplementary Information:**

The online version contains supplementary material available at 10.1186/s12967-022-03400-z.

## Background

Two oncogenic gammaherpesviruses, Kaposi sarcoma-associated herpesvirus (KSHV) and Epstein–Barr virus (EBV), are responsible for several types of tumors and hyperproliferative diseases. KSHV is the etiologic agent for Kaposi sarcoma (KS), primary effusion lymphoma (PEL), and multicentric Castleman’s disease (MCD), as well as KSHV inflammatory cytokine syndrome (KICS) [1, 2]. EBV is etiologically relevant to various human tumors including Burkitt’s lymphoma (BL), Hodgkin's lymphoma (HL), post-transplant lymphoproliferative disease (PT-LPD), undifferentiated nasopharyngeal carcinoma (NPC), and a distinct subset of gastric carcinomas (GaCa) [3]. While some of these tumors can be treated, there is an urgent need for new therapeutic modalities.

Both viruses utilize multiple strategies to evade the human immune system. One strategy is to inhibit the expression of immune surface molecules, such as major histocompatibility antigen class I (MHC-I), intracellular adhesion molecule 1 (ICAM-1), and B7-2, also called CD86. Two KSHV genes, K3 and K5, serve as E3 ubiquitin ligases and promote degradation of various cell immune surface molecules including MHC-I, ICAM-1, and B7-2 [4–7]. Also, KSHV-encoded latency-associated nuclear antigen (LANA) can inhibit MHC-I expression [8]. Multiple EBV genes including *BNLF2a, BGLF5, BILF1, BCRF1*, downregulate MHC-I expression by directly interfering with the HLA-I antigen presentation pathway [9–12]. EBV encoded viral interleukin-10 (vIL-10) inhibits not only MHC-I, but also ICAM-1 and B7 expression on monocytes [13]. Downregulation of MHC-I by these KSHV and EBV-encoded genes impairs antigen presentation to CD8+ T cells. Moreover, diminished ICAM-1 and B7-2 expression enables evasion of both T cell and natural killer (NK) cell immunity. This downregulation occurs in tumors caused by KSHV or EBV and makes the tumors relatively invisible to the immune system. These findings suggest that restoration of these surface immune markers could potentially enhance immune recognition and elimination of virus-infected tumor cells.

Cell cycle dysregulation is a hallmark of gammaherpesviruses-mediated oncogenesis, and cyclins are important for the survival of gammaherpesvirus-induced tumors [14]. Cyclin D2 has been shown to be crucial to the survival of KSHV-infected PELs [15]. KSHV LANA cooperates with the vCyclin-CDK6 kinase complex to facilitate latency and enhance cell proliferation [16, 17]. Cyclin D1 overexpression is required for EBV to stably infect nasopharyngeal epithelial cells [18]. EBV latent membrane protein-1 (LMP-1)-induced expression of cyclin D2 contributes to B cell transformation and uncontrolled cell proliferation [19]. All these observations underscore the role of cyclin D-CDK4/6 as a crucial regulator for gammaherpesvirus-mediated oncogenesis and further suggest that cyclin D-CDK4/6 could potentially serve as an immunomodulatory target for KSHV- or EBV-related tumors.

CDK4/6 inhibitors were developed to block cyclin D-CDK4/6 complex formation, preventing phosphorylation of Rb1 and resulting in cell cycle arrest from G1 to S phase [20]. Three inhibitors are now approved by the US Food and Drug Administration (FDA) for the treatment of breast cancer: abemaciclib (Abe), palbociclib (Pal), and ribociclib (Rib) [21]. A recent study showed that PEL cell lines require cyclin D2 expression and are highly sensitive to treatment with Pal in vitro [15].

Several studies have revealed that in addition to blocking the tumor cell cycle, CDK4/6 inhibitors can upregulate MHC-I expression in colon cancer cells [22–24]. Evidence was presented that this effect was mediated through enhanced expression of ERV3-1, leading to an interferon type III response that includes MHC-I upregulation. If CDK4/6 inhibitors could upregulate surface immune markers, this could potentially facilitate their activity against KSHV or EBV-induced tumors. However, we were concerned that such an effect may not be seen, since these tumors are chronically infected with gammaherpesviruses.

To explore the potential utility of CDK4/6 inhibitors in gammaherpesvirus-induced tumors, we investigated the effect of the 3 approved CDK4/6 inhibitors, Abe, Pal, and Rib on the proliferation of PEL cells, of KSHV-infected human umbilical endothelial cells (HUVEC), and of EBV-infected BL cells. In addition, we investigated their potential to reverse the downregulation of MHC-I, ICAM-1, and B7-2 in these tumor cells and the potential role of drug-induced changes in gammaherpesvirus and ERV3-1 expression on these effects.

## Materials and methods

### Cells and cell culture

BJAB cells, PEL cell lines JSC-1, BCBL-1, BC-1, and BC-2; and EBV cell lines Akata, Raji, and Daudi, were obtained and maintained as described previously [25, 26]. JSC-1, BC-1, and BC-2 are co-infected with EBV, while BCBL-1 is not. The KSHV-infected iSLK cell line (BAC16 strain) was kindly provided by Rolf Renne from the University of Florida and maintained in DMEM (Gibco) supplemented with 10% fetal bovine serum (HyClone), 1.0 μg/mL puromycin, 1.2 mg/mL hygromycin, and 250 μg/mL G418. HUVECs were purchased from Lonza and maintained in EGM-2 Bulletkit (Lonza) for up to 5 passages, with passages 3 to 5 used for experiments. All cells were maintained at 37 ℃ and 5% CO_2_ in Falcon cell culture flasks. For the treatment of cells with inhibitors, floating cells were seeded at 2 × 10^5^/mL and treated with CDK4/6 inhibitors at indicated concentrations for up to 3 days, while adherent cells were treated for up to 7 days.

### Test compounds

Abemaciclib (LY2835219) and palbociclib (PD-0332991) were purchased from Selleck Chemicals and dissolved in ethanol and water respectively at a stock concentration of 10 mM/mL. Ribociclib (LEE011, 10 mM/mL in DMSO) was purchased from MedChemExpress. All stocks were aliquoted and stored at − 20 °C.

### Preparation of KSHV stock, and de novo infection

For preparation of virus stock of KSHV-BAC16, which latently expresses green fluorescent protein (GFP), iSLK cells were treated with doxycycline (1 µg/mL; Thermo Fisher Scientific) and sodium butyrate (1 mM; Sigma Aldrich) for 3 d to induce lytic replication. Collected supernatants were cleared of debris with centrifugation at 500×*g* for 5 min, filtered (Rapid-Flow 0.45-µm filter; Thermo Fisher Scientific) and pelleted with ultracentrifugation (2.5 h at 4 °C at 50,000×*g* with SW 32 Ti; Beckman Coulter). Pellets were then resuspended with Dulbecco’s Modified Eagle Medium (DMEM) (Thermo Fisher Scientific). For de novo infection of HUVEC, cells were infected with diluted virus at a multiplicity of infection (MOI) of 15, as determined by LANA copy number, with 8 μg/ml polybrene. Virus supernatants were washed off after 8 h and replaced with fresh Endothelial Growth Media-2 (EGM2, Lonza Catalog #: CC-3162). HUVEC were refed every 2 days until harvested. Cells were visualized using ZOE fluorescent microscope (BioRad) and the percentage of infected cells were assessed as the percent of GFP+ cells using ImageJ software.

### Assay for viable cells

The relative number of viable cells was analyzed using CellTiter-Glo® Luminescent Cell Viability Assay kit (Promega). Briefly, 50 μL reagent was added to 50 μL cells in 96-well plate. Contents were mixed for 2 min on an orbital shaker and then incubated at room temperature for 10 min. Luminescence was recorded using a plate reader VICTOR™ X3 (PerkinElmer). The percentage of alive vs dead cells was assessed using trypan blue staining.

### Flow cytometry analysis and antibodies

Analysis of cells for surface marker expression was carried out as described previously [25]. Briefly, control and treated cells were incubated with 1:50 diluted PerCP/Cyanine5.5-labeled isotype antibodies or antibodies against HLA class I (A, B and C) (Cat. #:311420), ICAM-1 (Cat. #:353120), B7-2 (Cat. #:374216), or PD-L1 (Cat. #:329738) from BioLegend, for 40 min, and then were washed three times with FACS buffer (2% FBS, 1 mM EDTA, Ca/Mg^2+^-free phosphate buffered saline [PBS]), suspended in FACS buffer, and then analyzed with a flow cytometrycalibur™ Flow Cytometry system (BD Biosciences). Data were analyzed and visualized using FlowJo software (flowjo.com).

### T-cell activation assay

T-cell activation assays were performed using the T-cell activation bioassay IL-2 promoter kit (Promega, cat# J1651) as described previously [27]. Briefly, PEL cells were treated with the indicated concentrations of CDK4/6 inhibitors for 3 days, after which the cells were washed with PBS and mixed with T cell receptor (TCR)/CD3 Effector Cells (Jurkat T-cells expressing a luciferase reporter gene under IL-2 promoter) at a 2:1 ratio for BCBL-1 to Jurkat, and 1: 2 for Akata to Jurkat, and stimulated with various concentrations of anti-human CD3 monoclonal antibody (OKT3) from ThermoFisher Scientific (cat# 16-0037-81) in a 37 °C incubator for 6 h. The mix and incubation were done in triplicate in 96 well-plates containing 25 μL of target cells, 25 μL of Jurkat cells, and 25 μL of anti-CD3 antibody per well. Bio-Glo reagent was then added for a 10 min incubation at room temperature. The signal was captured using a Victor X3 multilabel plate reader (PerkinElmer). Wells containing cells but no Bio-Glo reagent served as background luminescence control. Luminescence data was plotted as a 4PL regression graph using GraphPad Prism software. Fold change in activation was calculated after subtracting signal obtained from Jurkat cells without co-stimulation by target cells from that obtained with co-stimulation by target cells.

### RT-qPCR

mRNA was extracted using the RNeasy kit (Qiagen). cDNA synthesis was performed using random primers with the High-Capacity cDNA Reverse Transcription Kit (ThermoFisher Scientific) on a T100 Thermal Cycler (Bio-Rad). To evaluate the mRNA level of host genes including *MHC-I, ICAM-1, B7-2,PD-L1, ACTB*, *DDX58*, *DNMT1*, *ERV3-1*, *IFIT1*, interferon(IFN)-λ2 (*IFNL2)*, *IFN-α, IFN-β, IFN-γ, NLRC5*, *OAS2*, and *STAT1*, and viral genes including KSHV-encoded open reading frame (*ORF)73* (LANA), *ORF50* (regulator of transcription activator [RTA]), *K2* (viral interleukin-6 [vIL6)], *ORF45*, and *ORF57*, and EBV-encoded *EBER2* and *BMRF1*, SYBR green qPCR assays were performed using the Applied Biosystems® SYBR® Green PCR Master Mix (ThermoFisher Scientific) on an ABI StepOnePlus real-time PCR system (ThermoFisher Scientific). Primers used are listed in Additional file 8. Relative mRNA expression levels were analyzed using the ΔΔCt method with genes coding for β-actin as the reference gene.

### Human IL-29/IL-28B DuoSet ELISA

Cell culture media was collected into 1.5 mL Eppendorf tubes and purified by centrifugation at 15,000*g* for 10 min. Supernatants were collected and analyzed by ELISA for IL28B and IL29 production using Human IL-29/IL-28B DuoSet ELISA kit (R&D, Cat # DY1598B-05) following the manufacturer’s instruction. Briefly, 100 μL/well sample or standards were added and incubated for 2 h. After 3 washes, 100 μL/well detection antibody were added and incubated for 2 h. After 3 washes, 100 μL/well Streptavidin-HRP was added to each well, and incubated avoiding light for 20 min at room temperature. Following another 3 washes, 100 μL/well Substrate Solution was added and incubated for 20 min at room temperature without direct light. Finally, 50 μL/well Stop Solution was added, and optical density was measured using a microplate reader set to 450 nm.

### Statistical analysis

Statistical analysis was performed using two-tailed student’s t-test (paired or unpaired where indicated) on experiments with 3 or more biological replicates. P-values less or equal to 0.05 were considered statistically significant. Asterisks indicate p values: *p < 0.05, **p < 0.01, ***p < 0.001, ****p < 0.0001.

## Results

### CDK4/6 inhibitors inhibit growth of KSHV- and EBV-infected cells

**Fig. 1:**
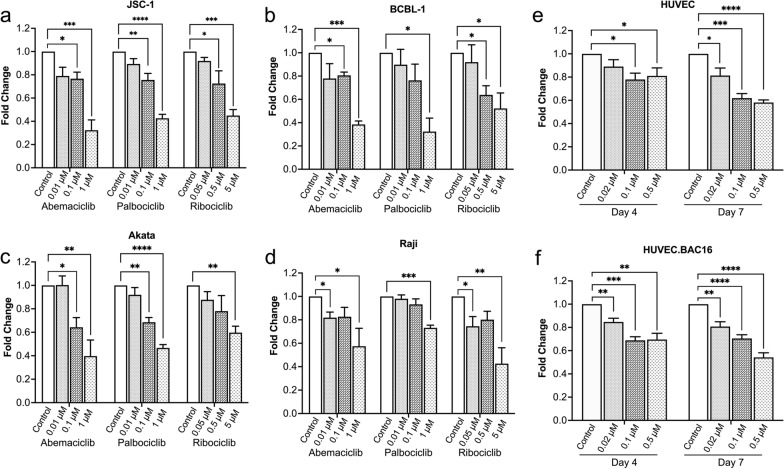
CDK4/6 inhibitors inhibit growth of KSHV^+^ cells and EBV^+^ cells. JSC-1 (**a**), BCBL-1 (**b**), Akata (**c**), and Raji (**d**) cells were treated in triplicate with tenfold increasing concentrations of Abe, Pal, or Rib, or with RPMI medium control for 72 h. HUVEC (**e**) and HUVEC.BAC16 (**f**) were treated in triplicate with fivefold increasing concentrations of abemaciclib, or with RPMI medium control for 4 or 7 days. The number of viable cells was assessed at different timepoints using CellTiter-Glo® Luminescent Cell Viability Assay. Shown are mean fold change in relative light units (RLU) from 3 experiments. Error bars indicate the standard deviations. Statistics were done using unpaired two-tailed *t*-test. Asterisks indicate p values: *p < 0.05, **p < 0.01, ***p < 0.001, ****p < 0.0001. Those without asterisks are not significant (p ≥ 0.05)

We first investigated the impact of CDK4/6 inhibitors on cell growth of various KSHV-infected cells including the PEL cell lines JSC-1 (Fig. 1a), BCBL-1 (Fig. 1b), BC-1 (Additional file 1a), and BC-2 (Additional file 1b), EBV-infected BL lines including Akata (Fig. 1c), Raji (Fig. 1d), and Daudi (Additional file 1c), and two EBV-negative BL lines including BJAB (Additional file 1d) and CA46 (Additional file 1e). All three CDK4/6 inhibitors exhibited dose-dependent inhibitory effect on cell growth for all the tested cell lines at day 3 (72 h) with about 30 to 70% inhibition observed at the highest doses tested (1 µM of Abe and Pal, or 5 µM Rib). We also tested the effects of Abe on the growth of uninfected HUVEC and KSHV-infected HUVEC cells. To produce KSHV-infected HUVEC, cells were exposed to concentrated viral stocks of KSHV.BAC16 using 15 viral DNA copy numbers per cell to reach around 85% GFP-positive rate by 24 h (Additional file 2). GFP is constitutively expressed in KSHV.BAC16-infected cells and GFP expression can be used to identify infected cells. Abe inhibited cell growth of KSHV-infected HUVEC starting at day 4 at concentrations of 0.02 µM or higher; by day 7, cell growth was inhibited about 50% at the highest dose tested (0.5 µM). In addition, Abe similarly inhibited the growth of uninfected HUVEC. The decrease in viable cell numbers after culture with CDK4/6 inhibitors was caused by a decrease in proliferation rather than an increase in cell death. After 3 days (B cells) or 4 days (uninfected or infected HUVEC cells) culture with Abe at doses that reduced cell numbers, there was no decrease in the percentage of viable cells as assessed by trypan blue exclusion (Additional file 3).

### CDK4/6 inhibitors increase MHC-I surface expression in KSHV- and EBV-infected cells

**Fig. 2:**
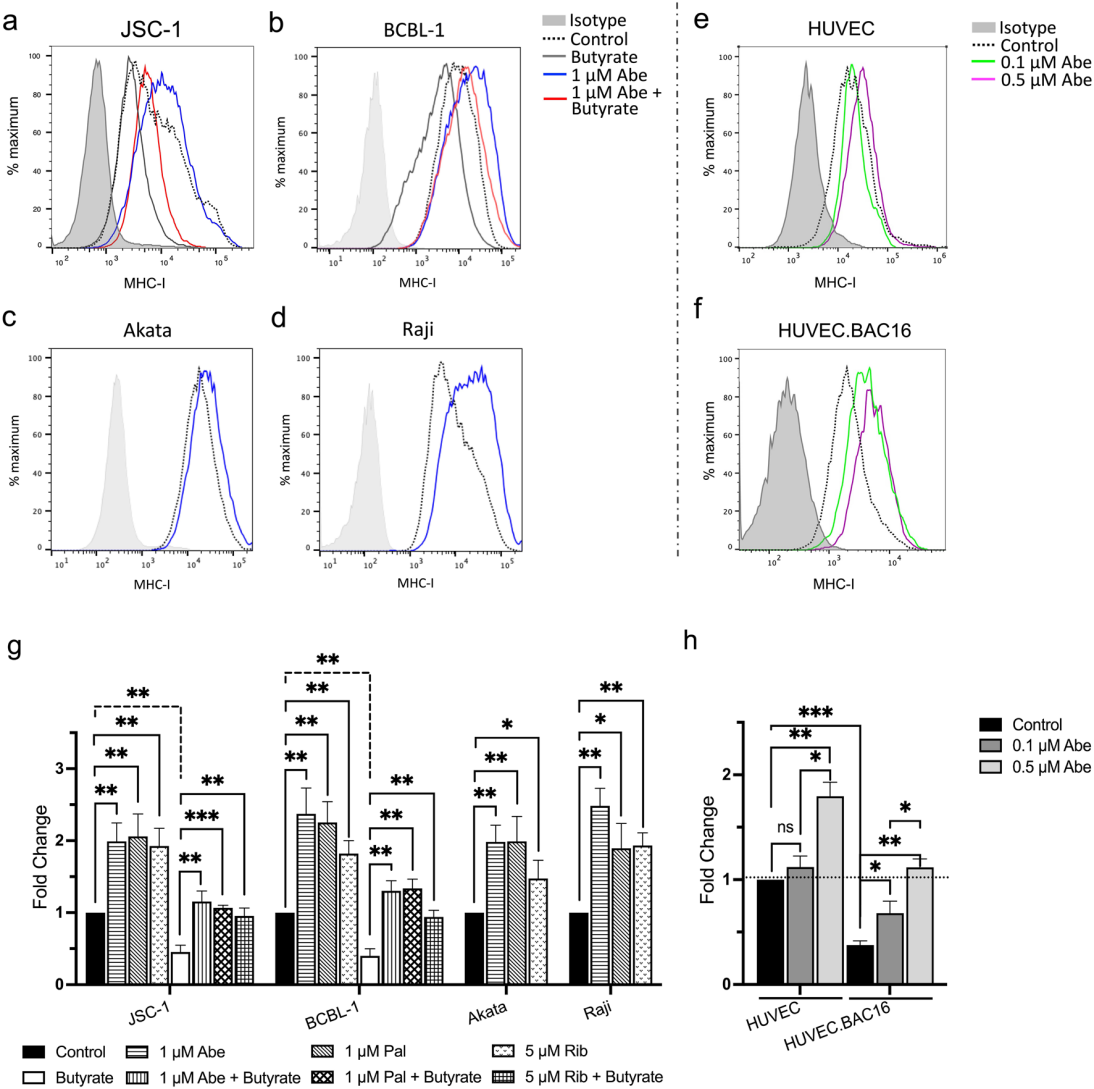
CDK4/6 inhibitors increase MHC-I surface expression on KSHV^+^ cells and EBV^+^ cells. Cells were treated either for 48 h (JSC-1 (**a**) and BCBL-1 (**b**)) or 72 h (Akata (**c**), and Raji (**d**)) with 1 μM Abe (**a–d**), 1 μM Pal, 5 μM Rib (**g–h**) or RPMI medium control. PEL cells (**a**, **b**) were then treated with sodium butyrate (0.3 mM) or RPMI medium control for another 24 h. The cells were then analyzed by flow cytometry for surface MHC-I expression. Endothelial cells (**e** and **f**) were treated for 4 days with 0.1 μM Abe, 0.5 μM Abe, or EGM-2 medium control and similarly analyzed for MHC-I expression. Results shown are one representative histograms of 3 separate experiments in **a**–**f**. Shown in **g** and **h** are the mean fold changes in median fluorescent intensity (MFI) and standard deviations from 3 experiments, as compared to the MHC-I in untreated control cells. Comparisons were performed using the unpaired two-tailed *t*-test. Asterisks indicate p values: *p < 0.05, **p < 0.01, ***p < 0.001, ns not significant

We next explored the effect of CDK4/6 inhibitors on the surface expression of MHC-I on KSHV- and EBV-infected cells. Several KSHV-encoded lytic genes including K3 and K5 can ubiquitinate MHC-I and reduce its surface expression. Consistent with this, MHC-I expression decreased to 37.5% of control for JSC-1 (Fig. 2a and g) and 31% for BCBL-1 (Fig. 2b and g) after lytic induction by butyrate. However, pretreatment of these cells with 1 μM Abe substantially prevented downregulation of MHC-I by butyrate (Fig. 2a, b, and g). Even without lytic induction, treatment of JSC-1 and BCBL-1 PEL lines with 1 µM Abe increased surface MHC-I expression (Fig. 2a, b). Treatment of two other PEL cell lines, BC-1 and BC-2, with 1 µM Abe, exhibited similar upregulation of MHC-I on the cell surface (Additional file 4, a-d). Abe also increased surface expression of MHC-I in the EBV-infected Akata and Raji cell lines (Fig. 2c, d). Figure 2g shows the mean and standard deviation from 3 experiments of the expression of MHC-I on JSC-1 and BCBL-1 PEL lines and on Akata and Raji EBV-infected BL lines. We further tested the effects of Pal and Rib, in addition to Abe, on MHC-I expression on JSC-1 and BCBL-1 PEL lines and on Akata and Raji EBV-infected BL lines. As can be seen in Fig. 2g, all three CDK4/6 inhibitors increased MHC-I expression on PEL lines induced to lytic replication as well as uninduced lines. Moreover, all three drugs enhanced MHC-I expression on Akata and Raji BL lines. EBV-uninfected BJAB a Burkitt’s lymphoma B cell line, also exhibited an increased MHC-I when treated with 1 µM Abe, although it was less than that of the virus-infected lines(Additional file 4e, f).

We also tested Abe’s effect on MHC-I expression in KSHV-infected and uninfected HUVEC (Fig. 2e, f, and h). Cells were infected with KSHV.BAC16 to obtain about 85% cells expressing GFP. As seen in Fig. 2h, while both 0.1 and 0.5 µM Abe induced significant and dose-dependent increases in MHC-I on KSHV-infected HUVEC, only 0.5 µM Abe induced a small increase of MHC-I in uninfected HUVEC.

### CDK4/6 inhibitors increase ICAM-1, B7-2 and PD-L1 surface expression in KSHV- and EBV-infected cells

**Fig. 3:**
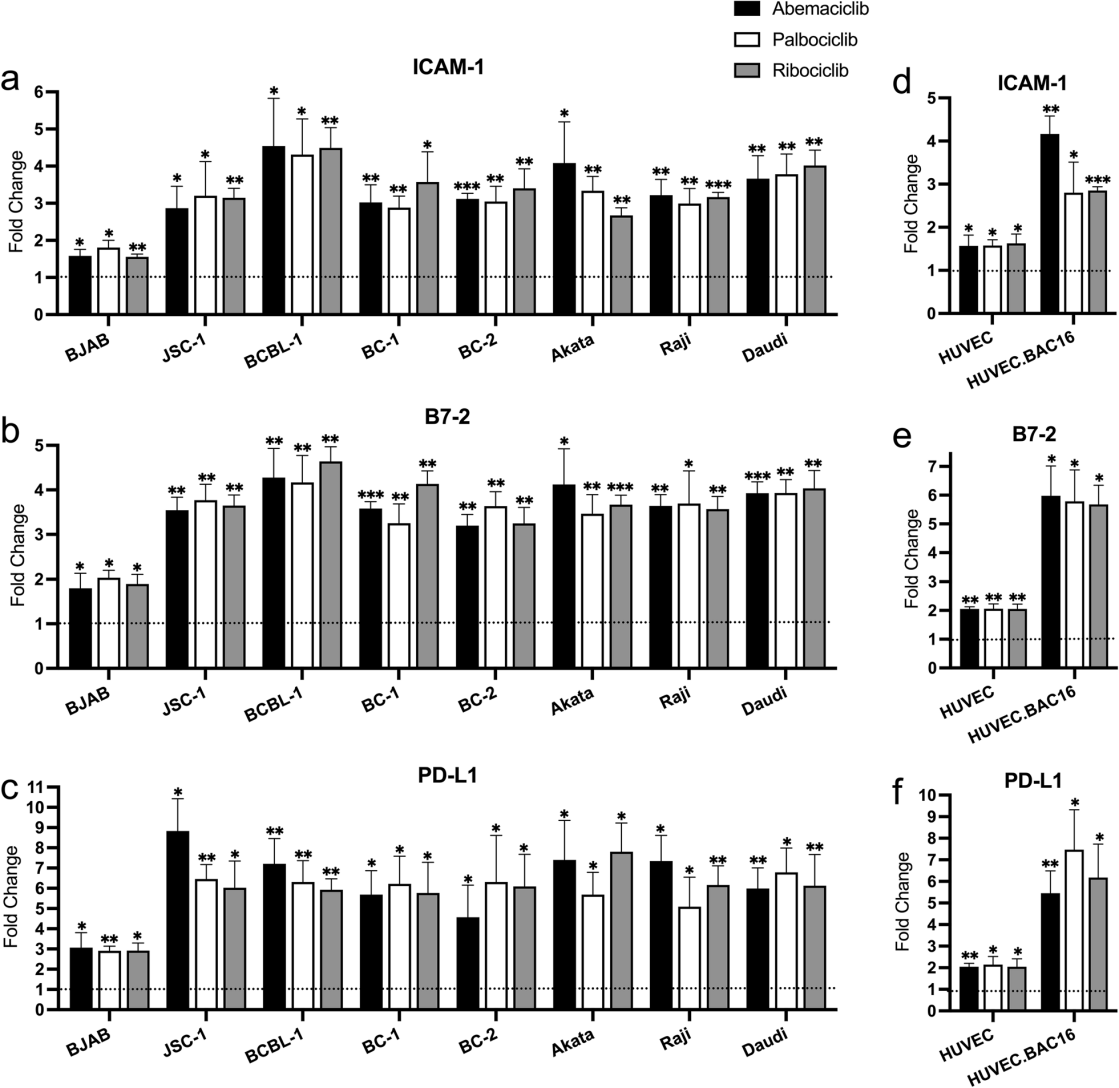
CDK4/6 inhibitors increase cell surface expression of ICAM-1, B7-2, and PD-L1 in KSHV- and EBV- uninfected and infected cells. BJAB, JSC-1, BCBL-1, BC-1, BC-2, Akata, Raji, and Daudi cells were treated with 1 μM Abe, 1 μM Pal, 5 μM Rib, or RPMI medium control for 72 h. HUVEC and HUVEC.BAC16 cells were treated with 0.5 μM Abe, 0.5 μM Pal, 2.5 μM Rib, or EGM-2 medium control for 4 days. Surface expression of ICAM-1, B7-2 and PD-L for the lymphoma cells (**a**–**c**) and endothelial cells (**d**–**f**) were analyzed by flow cytometry. Results shown are mean fold changes relative to untreated cells from 3 experiments. Statistics were done using unpaired two-tailed *t*-test. Asterisks indicate p values: *p < 0.05, **p < 0.01, ***p < 0.001

KSHV and EBV also downregulate surface expression of ICAM-1 and B7-2 [6, 13], which are important for T-cell and NK-cell activation and effector function. We assessed the effects of CDK4/6 inhibitors on surface expression of ICAM-1 and B7-2, as well as PD-L1 on virus-uninfected BJAB cells and the same set of virus-infected PEL and BL cell lines (Fig. 3; Additional file 5). BJAB, PEL cells and EBV-infected BL cells were treated with 1 μM Abe, 1 μM Pal, or 5 μM Rib for 3 days, while uninfected and KSHV-infected HUVEC were treated with 0.5 μM Abe, 0.5 μM Pal, or 2.5 μM Rib for 4 days. Cells were then analyzed using flow cytometry. All the tested cell lines exhibited significant increases in ICAM-1 and B7-2 surface expression in response to all 3 CDK4/6 inhibitors although the virus-infected lines showed bigger increases compared to the virus-negative BJAB line. The average fold increase for ICAM-1 in KSHV and EBV-infected cells ranged from 2.5 to 4.5 fold (Fig. 3a), and for B7-2 from 3.2 to 6.0 fold (Fig. 3b). In addition, all 3 CDK4/6 inhibitors increased expression of PD-L1 from 4.2 to 8.3 fold (Fig. 3c) in the virus-infected lines. Virus-uninfected BJAB cells exposed to the drugs had a substantially smaller increase in the surface markers. We also tested the effects of the drugs in uninfected and infected HUVEC cells. While there was a relatively small increase (1.5 for ICAM-1, 2.0 for B7-2 and PD-L1) in uninfected HUVEC, there was a more substantial increase (2.7 to 4.2 for ICAM-1, 25.4 to 5.8 for B7-2, and 5.2 to 7.2 for PD-L1) in expression of all 3 markers in KSHV-infected HUVEC (Fig. 3d–f, Additional file 6).

### CDK4/6 inhibitors increase mRNA expression of MHC-I, ICAM-1, B7-2 and PD-L1 in KSHV- and EBV-infected cells

**Fig. 4:**
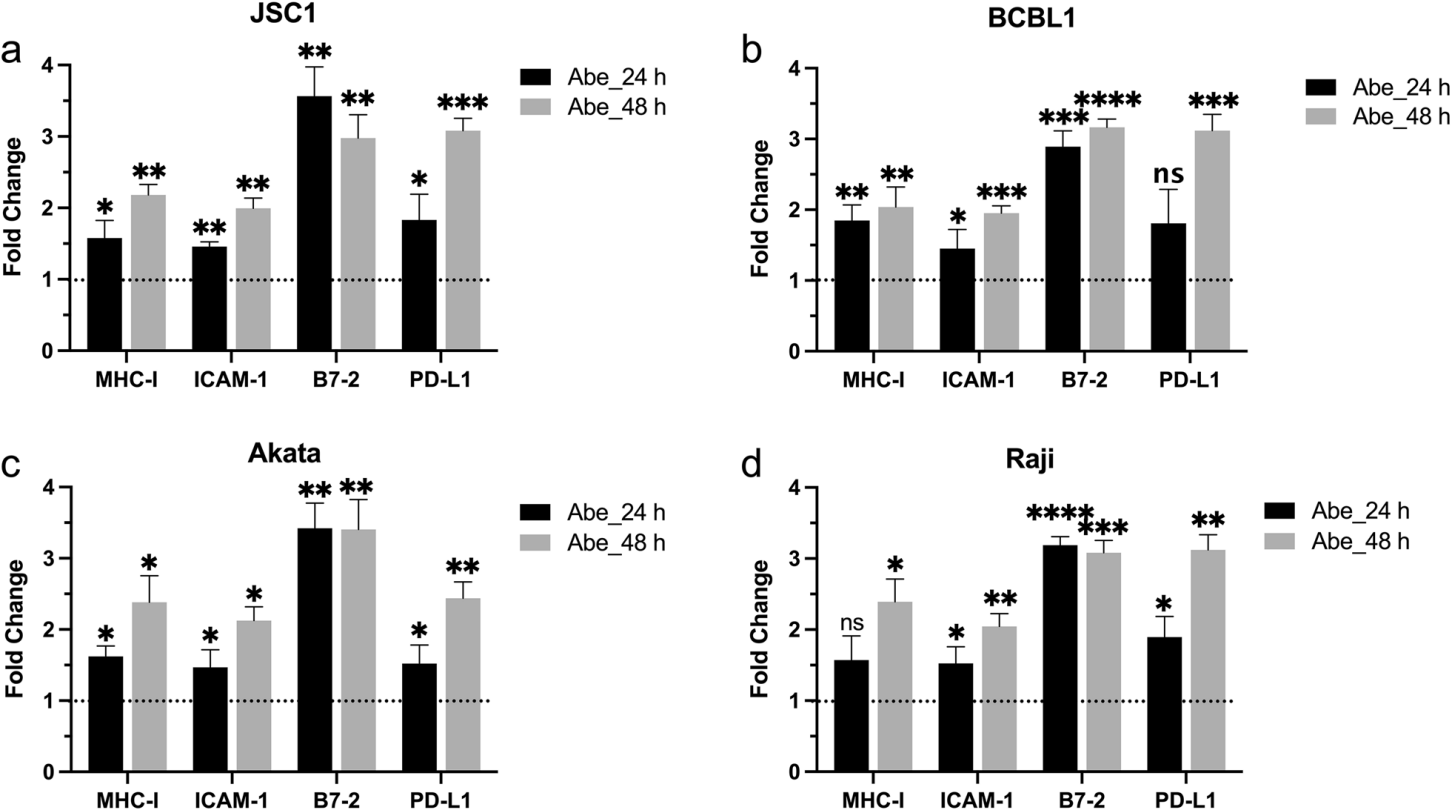
CDK4/6 inhibitors increase mRNA expression of MHC-I, ICAM-1, B7-2, and PD-L1 in KSHV^+^ cells and EBV^+^ cells. JSC-1 (**a**), BCBL-1 (**b**), Akata (**c**), and Raji (**d**) cells were treated with 1 μM abemaciclib, or with RPMI medium control for 24 h or 48 h. Total RNA was extracted and analyzed for mRNA expression of MHC-I, ICAM-1, B7-2 and PD-L1 using realtime qPCR. Results shown are mean fold changes relative to untreated cells from 3 experiments. Error bars represent standard deviations from the 3 experiments

We further evaluated the impact of CDK4/6 inhibitors on the expression of mRNA for these surface markers in JSC-1 (Fig. 4a), BCBL-1 (Fig. 4b), Akata (Fig. 4c), and Raji (Fig. 4d) cells. Cells were cultivated with 1 μM Abe or solvent control (diluted ethanol in medium) for 24 h or 48 h and the total RNA was then extracted for qPCR analysis. As the result shows, mRNA expression of all these 4 genes were significantly increased at both time points after treatment, with an average fold increase of 1.6–1.8 for MHC-I, 1.4–1.5 for ICAM-1, and 2.8–3.4 for B7-2 at 24 h, and 2.0–2.4 for MHC-I, 1.9–2.2 for ICAM-1, and 2.8–3.3 for B7-2 at 48 h. Also, mRNA for PD-L1 increased significantly from 1.5 to 1.8 fold at 24 h, and 2.4 to threefold at 48 h for all cells except for BCBL-1 cells at 24 h. These results provide evidence that the increased expression of the surface markers by Abe was at least in part from increased transcription of the genes.

### CDK4/6 inhibitor-treatment of PEL and EBV-infected BL cells enhances T-cell activation by these lines

**Fig. 5:**
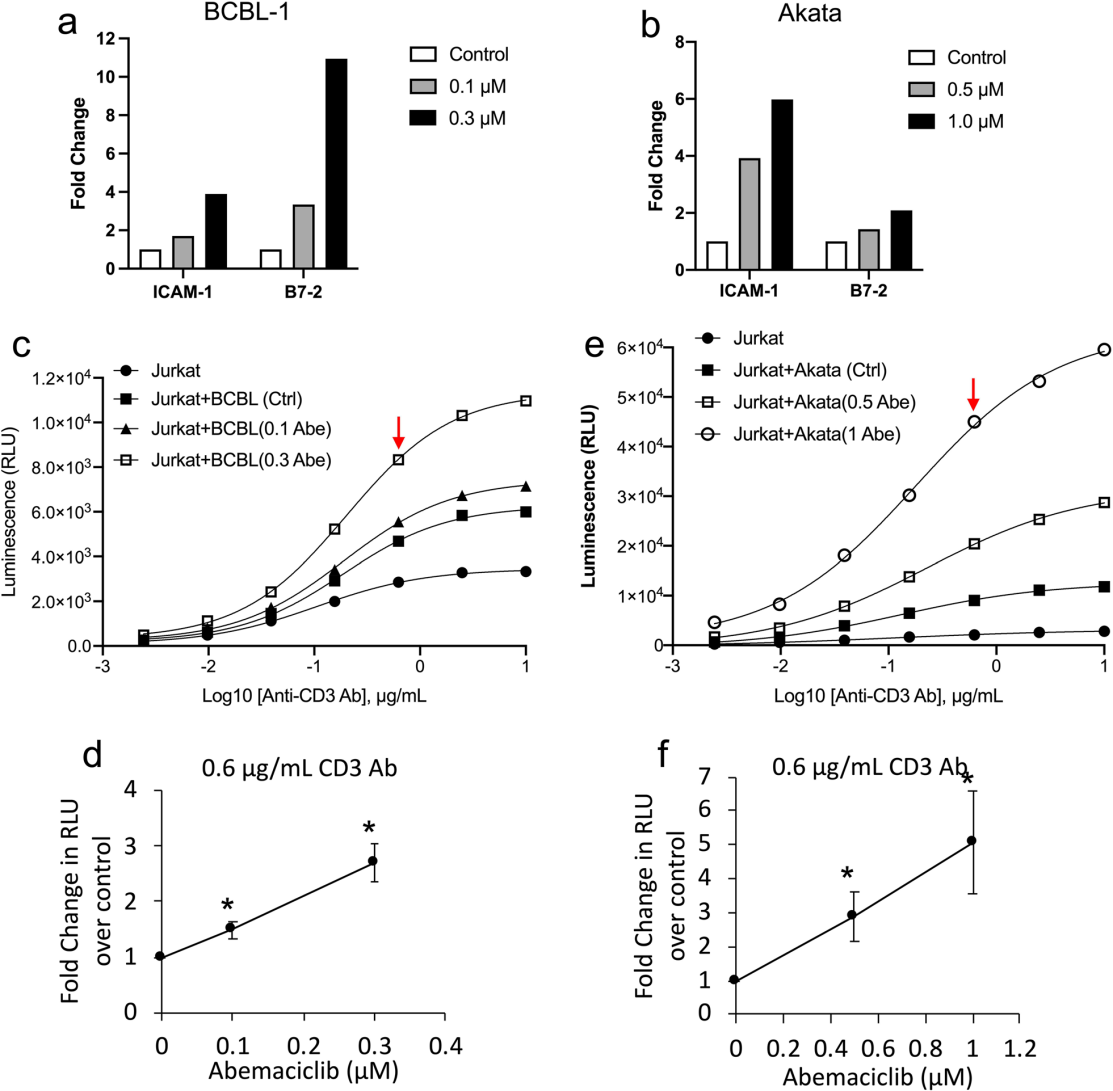
CDK4/6 inhibitor-treated KSHV^+^ cells and EBV^+^ cells express increased ICAM-1 and B7-2 and induce increased T-cell activation. **a**, **b** Surface expression of ICAM-1 and B7-2 in BCBL-1 cells treated with 0.1 μM or 0.3 μM Abe (**a**) and Akata cells treated with 0.5 μM or 1 μM Abe (**b**) for 72 h. Cells were then harvested, and stained with PerCP/Cy5.5-conjugated IgG isotype control, anti-ICAM-1, or anti-B7-2 antibodies and analyzed using flow cytometry. Shown are fold changes in median fluorescent intensity (MFI) of one experiment of Abe-treated cells over untreated control cells. **c**–**f** T-cell activation in BCBL-1 (**c** and **d**) and Akata (**e** and **f**) cells in response to Abe treatment in the same experiment analyzed in panels **a** and **b**. Treated cells were washed with PBS to remove Abe and coincubated for 6 h with Jurkat cells expressing luciferase under the control of the IL-2 promoter at a 2:1 (BCBL-1) or 1:2 (Akata) target to effector ratio in the presence of anti-human CD3 antibody to measure Jurkat T-cell activation. Activation of the Jurkat T-cells are shown as relative light units (RLU) (**c** and **e**). Average fold changes in RLU from Jurkat cells coincubated with Abe-treated BCBL-1 and Akata cells over those coincubated with ethanol control (0 μM Abe) treated cells in the presence of 0.6 μg/mL anti CD3 antibody are shown in **d** and **f**. Error bars represent standard deviations from 3 independent experiments. Statistically significant differences (*p ≤ 0.05, paired 2-tailed t-test) between control and Abe-treated cells are indicated

We next sought to assess whether these CDK4/6 inhibitor-treated cells would enhance T-cell activation through increased expression of co-stimulatory molecules. As in previous experiments, 3-days of treatment of BCBL-1 PEL cells and Akata BL cells by Abe upregulated surface expression of ICAM-1 and B7-2 (Fig. 5a, b). T-cell activation induced by Abe was assessed in aliquots of the same cell culture using Jurkat cells expressing a luciferase reporter gene under control of the IL-2 promoter as the effector cells, and anti-CD3 antibody was used to activate these cells. Both BCBL-1 and Akata cells cultured in the absence of CDK4/6 inhibitors increased Jurkat T-cell activation above the baseline (Fig. 5c, e). However, Abe-treated BCBL-1 and Akata cells further stimulated the Jurkat T-cell activation compared to the control untreated cells in a dose-dependent manner (Fig. 5c, e). When co-stimulated with 0.6 μg/mL anti-CD3 antibody (red arrow in Fig. 5c, d), the mean increases in T-cell activations induced by 0.3 μM Abe-treated BCBL-1 cells and 1 μM Abe-treated Akata cells were 2.6 fold and fivefold over control-treated cells, respectively.

### CDK4/6 inhibitors induce increased expression of KSHV and EBV genes, as well as ERV-3

Abe has been shown to suppress expression of DNA methyltransferases which in turn leads to increased expression of the endogenous retrovirus ERV3-1, and it has been suggested that this may be a mechanism for the increased expression of MHC-I [23, 28]. In particular, it has been suggested that the ERV3-1 stimulates interferon type III which in turn leads to increased expression of interferon-induced genes including MHC-I. We were interested to see if such a mechanism might apply in the cell lines studied here, which were infected with an exogenous virus and might thus have high basal levels of interferon expression that would not be increased by ERV3-1. We were also interested to explore the possibility that Abe-induced expression of KSHV and/or EBV genes might contribute to the effect [29–31].

**Fig. 6:**
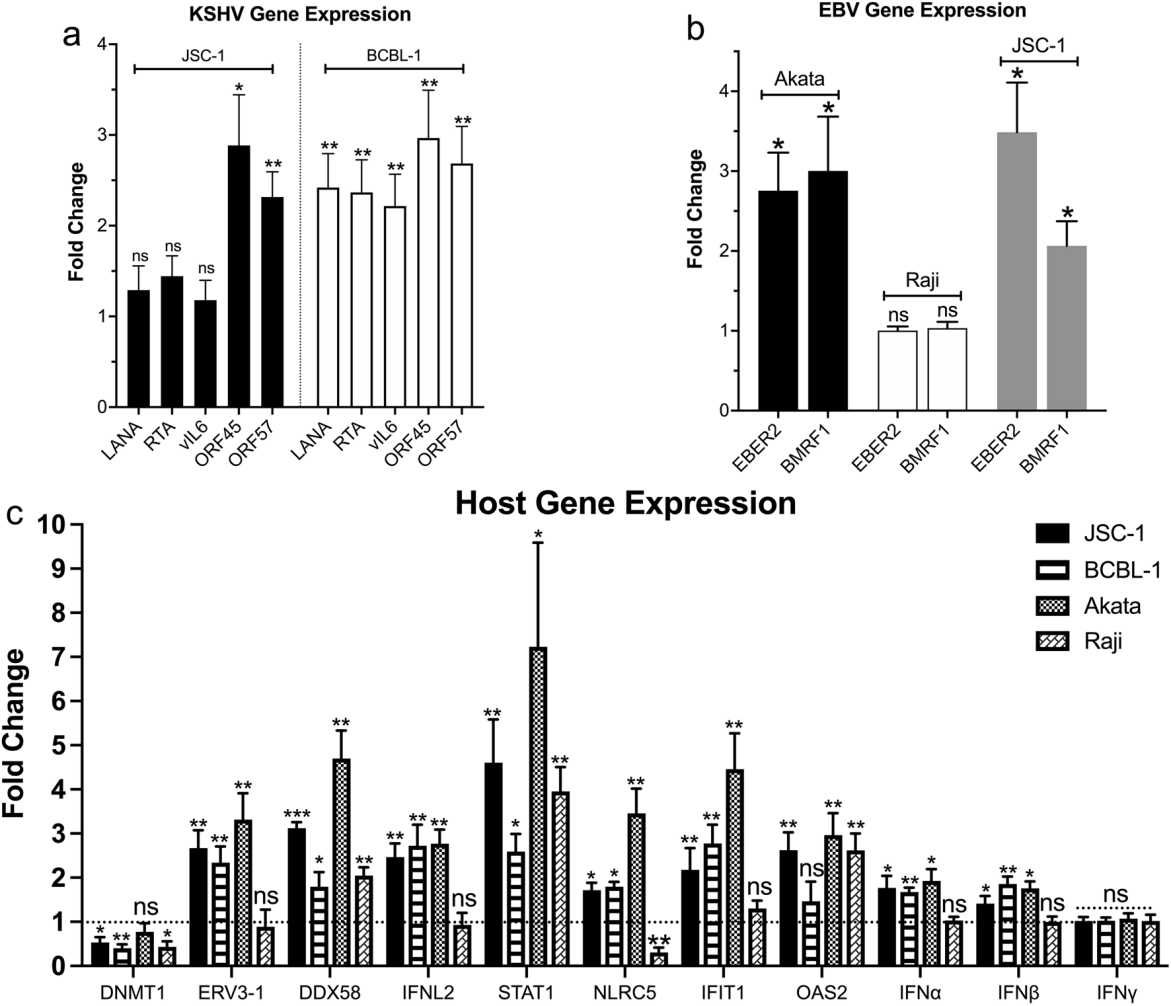
Effect of CDK4/6 inhibitors on viral and interferon-related cellular genes. KSHV genes (**a**), EBV genes (**b**), and cellular genes related to interferon pathway (IFN alpha, IFNbeta, IFNgamma, ERV3-1, *DNMT1*, *DDX58, IFNL2*, and selected interferon-stimulated genes) (**c**) in cells treated either with ethanol control (0 μM Abe) or 1 μM Abe for 24 h. Total RNA was extracted from whole cell lysates and expression of specific genes was assayed by RT-qPCR. mRNA levels were normalized to β-Actin and compared to those in control cells. Shown are average fold changes of mRNA levels in Abe-treated cells relative to ethanol-treated control cells from 3 independent experiments. Error bars represent standard deviations from 3 independent experiments. Statistically significant differences (*p ≤ 0.05, **p ≤ 0.01, ns not significant, paired 2-tailed t-test) between control and Abe-treated cells are indicated

To this end, we evaluated the mRNA expression levels of selected latent and lytic gammaherpesvirus genes, DNA methyltransferase 1 (*DNMT1),* the endogenous retrovirus ERV3-1, *IFN-α, IFN-β,* IFN-γ, *IFN-λ2*, and selected interferon-induced genes after treatment of KSHV- and EBV-infected tumor lines with 1 µM Abe for 24 h. After Abe treatment, the KSHV lytic genes ORF45 and ORF57 were significantly increased in both BCBL-1 and JSC-1 PEL lines (Fig. 6a). In addition, the lytic genes K2 (vIL-6), ORF50 (encoding (RTA)), and ORF73 (encoding latency-associated nuclear antigen (LANA)) were upregulated in BCBL-1 cells, but not JSC-1 cells. Also, treatment with 1 µM Abe for 24 h upregulated expression of *EBER2*, the most abundant EBV latent transcript, and *BMRF1*, a lytic gene, in Akata cells and JSC-1 cells, but not in Raji cells (Fig. 6b). Thus, Abe treatment variably increased expression of gammaherpesvirus latent and lytic genes in these cell lines.

*DNMT1,* which suppresses expression of endogenous retroviruses and has been reported to suppress of certain gammaherpesvirus genes [32], was substantially downregulated in both JSC-1 and BCBL-1 PEL cells and in Raji EBV-infected BL cells after treatment with 1 µM Abe; there was also a trend towards a decrease in Akata cells although the change was not significant (Fig. 6c). Expression of the endogenous retroviral gene *ERV3-1*, which is suppressed by *DNMT1*, was significantly upregulated in all the tested lines except Raji (Fig. 6c). In addition, the dsRNA sensor *DDX58*, which senses endogenous retroviruses and stimulates IFN signaling, was also upregulated in all four cell lines (Fig. 6c). We next looked specifically at expression of interferons. We found that *IFNL2, IFN-α*, and *IFN-β*, but not IFN-γ mRNA expression was increased in three of the lines tested (JSC-1, BCBL-1, and Akata), but not in Raji cells (Fig. 6c). Moreover, using an ELISA kit that measures IL28B plus IL29, two other members of the interferon family, we found an increase in the supernatants of JSC-1 and BCBL-1 cells after treatment with 1 μM Abe for 3 days (Additional file 7).

To evaluate the downstream effects of enhanced interferon activity, we measured the IFN-sensitive transcription factors *STAT1* and *NLRC5*, as well as two IFN-stimulated genes, *IFIT1* and *OAS2* in these cells. All these 4 genes exhibited significantly enhanced expression in JSC-1, BCBL-1, and Akata cells after exposure to Abe (Fig. 6c). However, Raji was again somewhat of an outlier in that *IFIT1* was not significantly changed and *NLRC5* was decreased (Fig. 6c).

## Discussion

**Fig. 7:**
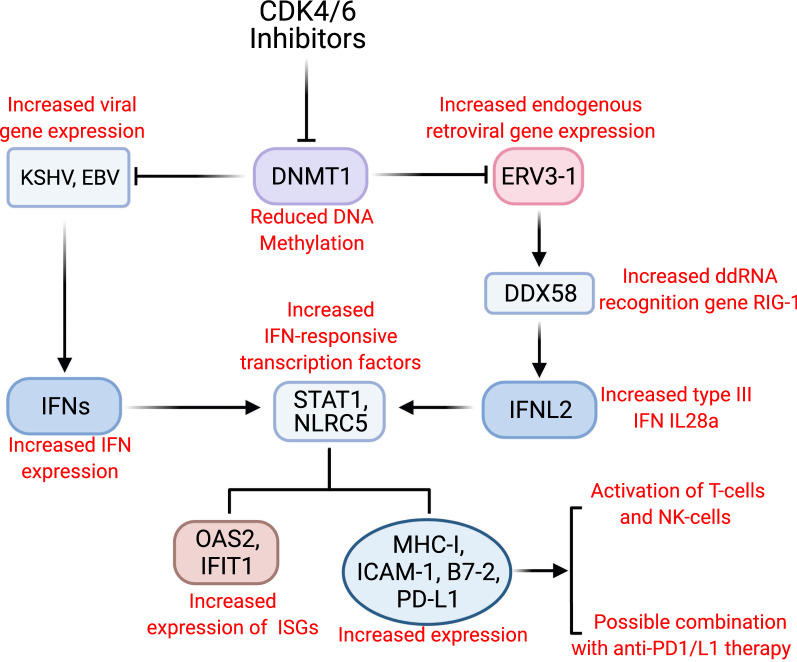
Schematic of proposed mechanism for CDK4/6 inhibitors’ effects on surface immune molecules in KSHV^+^ cells and EBV^+^ cells. In addition to direct inhibition of tumor cell proliferation, CDK4/6 inhibitors downregulate DNA methyltransferase 1, which activates both ERVs and certain KSHV/EBV genes. The DNA and RNA viral elements stimulate IFNs, which activate transcription factors including STAT1 and NLRC5. These genes in turn transactivate the expression of ISGs and immune surface molecules including MHC-I, ICAM-1, B7-2, and PD-L1. These surface molecules enable killing of the tumor by binding to receptors on T cells and potentially NK cells too. Because expression of PD-L1 is also enhanced, the results suggest that it may be worth testing CDK4/6 inhibitors with anti-PD1/L1 therapy

Several studies have shown that in addition to a direct effect on cancer cells, CDK4/6 inhibitors can enhance expression of surface MHC-I on certain tumors, thus making the cells more visible to the immune system [9–12, 22–24]. In this report, we extend these findings by showing that pharmacological inhibition of CDK4/6 can enhance expression of MHC-I in cells infected by two oncogenic herpesviruses, KSHV and/or EBV. Moreover, we show that these inhibitors also upregulate expression of ICAM-1 and B7-2 in the infected tumor cells, which can enable NK killing and enhance sensitization of T-cells to the tumor cells. We further found that the increased surface expression induced by Abe was at least in part due to increased mRNA expression of these genes. Finally, we demonstrate that Abe enhances T cell activation induced by PEL cell lines. A schematic figure outlining the proposed mechanism for the upregulation of surface immune markers by CDK4/6 inhibitors in EBV and/or KSHV-infected cells and subsequent activation of T cells and NK cells is presented in Fig. 7.

It has been shown that the KSHV vCyclin/CDK6 complex is constitutively activated in KSHV-infected cells [33]. Also, PEL cells are highly dependent on cyclin D2, which is required for cell cycle G1/S transition by complex with CDK4 or CDK6 [15]. With regard to EBV, the viral-encoded protein LMP-1 induces the expression of cyclin D2 to promote uncontrolled cell proliferation in EBV-positive Burkitt’s lymphoma cell lines [19]. These data suggest that CDK4/6 inhibitors may suppress growth of KSHV- and/or EBV-induced tumors, and Manzano et al. have shown that the CDK4/6 inhibitor Pal can lead to a striking G1 arrest in BCBL-1 and BC-3 PEL cells [15]. Our results extend this observation and show that Pal and two other CDK4/6 inhibitors can also suppress growth of EBV-infected tumor cells as well as KSHV-infected HUVEC cells. Interestingly, there was no significant difference in the degree of growth inhibition observed between EBV-infected Burkitt’s cells and the control EBV-uninfected Burkitt B cells (Fig. 1 and Fig. S1), suggesting that this growth inhibitory effect of CDK4/6 inhibitors did not require viral components.

Virus-induced tumors are potentially quite susceptible to immunologic control since they express virally encoded foreign proteins. However, oncogenic viruses have evolved potent mechanisms to suppress expression of surface immune markers, thus enabling infected cells and the virus-induced tumors to evade detection by the immune system [4–8, 13]. Approaches to reverse this downregulation might thus be important means of controlling these tumors. Recently, several studies indicated that in addition to inhibiting cell proliferation, CDK4/6 inhibitors may upregulate genes encoding MHC-I and the antigen presentation pathway in breast or colon tumors [23], or alter the tumor microenvironment by suppression of regulatory T cell proliferation [23, 34, 35], or enhanced activation of tumor-infiltrating T cells [22, 24]. These studies also provided evidence that the upregulation of MHC-I by CDK4/6 inhibitors was the result of degradation of DNMT1, leading to activation of the endogenous retroviruses (ERVs) and then to activation of interferons and interferon-induced genes [23]. Since the chronic gammaherpesvirus infection of KSHV- and EBV-induced tumors may already provide stimulation of interferon, we wondered whether a similar upregulation of MHC-I would be seen with CDK4/6 inhibitors. In fact, we found a robust upregulation of MHC-I in all the KSHV- and EBV-associated tumors, as well as KSHV-infected HUVEC cells. In addition, we found that these drugs substantially upregulated ICAM-1 and B7-2.

The effect seen here of ICAM-1 and B7-2 upregulation by CDK4/6 inhibitors is noteworthy. ICAM-1 and B7-2 are important co-factors for both T cell and NK cell killing [25, 36–38]. MHC-I expression is important for T cell killing, while MHC-I downregulation will generally activate NK cell killing in the face of ICAM-1 and B7-2 expression. However, downregulation of all three surface proteins, as occurs in gammaherpesvirus-infected cells, may enable escape of both T cell and NK cell killing. By upregulating both ICAM-1 and B7-2, along with MHC-I, CDK4/6 inhibitors thus render the tumor cells susceptible both to T cell killing and to certain types of NK cell killing. This conclusion is bolstered by the observation of enhanced T cell activity seen here with CDK4/6 treatment.

It should be noted that our results also show that CDK4/6 inhibitors can enhance expression of PD-L1, which might suppress the immune response induced by expression of other immune surface markers. Antibodies against PD-1 or PD-L1 have recently been shown to reverse immunologic suppression mediated by PD-1/PD-L1 and have potent activity against certain tumors that express new epitopes [39]. The observed increase of PD-L1 in gammaherpervirus-induced cells induced by Abe suggests that CDK4/6 inhibitors may be most effective immunologically if administered with anti-PD-1 or anti-PD-L1 therapy. In this regard, recent mouse tumor studies have shown that such therapy can augment CDK4/6-induced tumor control [22–24].

Previous studies of the effect of CDK4/6 inhibitors on MHC-I have provided evidence that the upregulation may be the result of DNMT1 degradation, leading to activation of endogenous retroviruses and subsequent interferon and interferon-induced gene activation [23, 40]. We wondered if a similar mechanism may apply in cells infected with gammaherpesviruses. We found that Abe does inhibit DNMT-1 and that this inhibition was associated with activation of the endogenous retrovirus ERV3-1, dsRNA sensor RIG-I, IFN-α, IFN-β, type III interferon IFN-λ2 (IL28A), IFN-sensitive transcription factors including STAT1 and NLRC5, and interferon-stimulated genes like IFIT1 and OAS2. NLRC5 is transcriptionally activated by STAT1, which can be induced by IFNα [41]. Since NLRC5 transactivates MHC-I [42], the elevated expression of STAT1and NLRC5 contributes to MHC-I overexpression. STAT1 also upregulates ICAM-1 and PD-L1 [43, 44]. Although studies have shown that IFN type I upregulates B7-2 [45, 46], whether the upregulation of B7-2 is through the same mechanism is still an unsolved puzzle. Interestingly, we also observed an increased expression of both latent and lytic KSHV and EBV viral gene mRNA expression, although the elevation observed (which ranged from 1.3-fold to 3.4-fold) varied among cell lines and was relatively small. It is unclear at this time whether ERV activation, activation of gammaherpesvirus genes, or both may contribute to the upregulation of surface markers in KSHV- or EBV-infected cells, and additional studies will be needed to further clarify the mechanism for these effects.

Our laboratory has previously shown that the immunomodulatory drug pomalidomide (Pom) also upregulates the immune surface molecules including MHC-I, ICAM-1, and B7-2 in a range of KSHV infected PEL and EBV-infected BL cells [25–27]. Pom has been shown to be clinically effective against KS and is in fact now approved for this indication [47], but it remains unclear if Pom upregulates these molecules in endothelial cells. Also, the combination of Pom plus pembrolizumab, an anti-PD-1 antibody, has been shown to be active in some patients with refractory EBV + lymphoma. As seen here, CDK4/6 inhibitors upregulate these molecules not only in KSHV + or EBV + lymphatic cells, but also in KSHV-infected endothelial cells. This indicates that CDK4/6 inhibitors might be worth testing for possible activity against KS. To explore this possibility, our group has initiated a clinical trial to test Abe in patients with KS (NCT04941274). Also, there is recent evidence that virus-induced tumors can be sensitive to anti-PD1 or anti-PD-L1 therapy, probably because they express foreign (viral encoded) proteins and because viruses often upregulate PD-L1 [48, 49]. Given that CDK4/6 inhibitors also upregulate PD-L1, it may be worth exploring the use of these drugs with anti-PD-1/PD-L1 therapy against virus-induced tumors in the future, although any benefits would have to be weighed against the potential for enhanced toxicity.

## Conclusion

In summary, CDK4/6 inhibitors are shown here to inhibit proliferation of PEL, KSHV-infected endothelial cells, and EBV+ Burkitt’s lymphoma cells and also to reverse virus-induced suppression of MHC-I, ICAM-1, and B7-2 on these cells. Treated cells were sensitized to T-cell killing probably due to the enhanced expression of these surface markers including ICAM-1 and B7-2. Gammaherpesviruses have evolved a variety of mechanisms to downregulate expression of these surface markers, thus rendering KSHV- and EBV-infected tumors relatively invisible to the immune system. By reversing this effect, CDK4/6 inhibitors may promote the immunologic control of gammaherpesvirus-induced tumors in addition to their direct effects on tumor cell proliferation.

## Supplementary Information

**Additional file 1. **CDK4/6 inhibitors inhibit growth of a variety of PEL and BL cell lines, BC-1 (a), BC-2 (b), Daudi (c), BJAB (d), and CA46 (e) were treated in triplicate with indicated concentrations of abemaciclib, or with RPMI medium control for 1, 2, and 3 days (except BJAB and CA46 cells that were treated only for 3 days). The number of viable cells was assessed using CellTiter-Glo® Luminescent Cell Viability Assay. Shown is the data from one representative experiment. Error bars indicate the standard deviations from 3 technical replicates.

**Additional file 2. **GFP expression in HUVEC infected by KSHV.BAC16. Cells were infected with diluted virus at a multiplicity of infection (MOI) of 15, as determined by LANA copy number, with 8 μg/ml polybrene. Virus supernatants were washed off after 8 h and replaced with fresh medium. The GFP signal was captured using the ZOE Fluorescent Cell Imager at 24 h post-infection. Shown are the brightfield view on the left and the GFP signal of the same field on the right.

**Additional file 3. **Abe reduces cell numbers but not the viability of KSHV^+^ cells and EBV^+^ cells. Cells were cultured in the absence or presence of indicated concentrations of Abe. After 3 days (JSC-1, BCBL-1, Akata and Raji) or 4 days (HUVEC and HUVEC.BAC.16), the number of viable cells was assessed using CellTiter-Glo® Luminescent Cell Viability Assay and the percentage of alive vs dead cells was assessed using trypan blue staining. Number of live cells and % viability (percentage of cells that are alive) for JSC-1 (a), BCBL-1 (b), Akata (c), Raji (d), HUVEC (e) and HUVEC.BAC16 (f) were calculated and presented as % of control-treated cells. Shown are the means from 3 separate experiments. Error bars indicate the standard deviations. Statistics were done using unpaired two-tailed *t*-test. Asterisks indicate p values: *p < 0.05, **p < 0.01, ***p < 0.001. Those without asterisks are all not significant (p ≥ 0.05).

**Additional file 4. ** Effects of Abe on MHC-I surface expression in BC-1 and BC-2 PEL lines and BJAB, an EBV-uninfected BL cell lines. Cells were treated either for 48 h (BC-1 (a and b) and BC-2 (c and d)) or 72 h (BJAB (e and f)), with 1 μM Abe or RPMI medium control. PEL cells (a-d) were then treated with sodium butyrate (0.3 mM) for another 24 h. All the cells were then analyzed by flow cytometry for surface MHC-I expression. Figures a, c, and e show a representative experiment. Figures b, d, and f show the mean fold change of MHC-I expression in BC-1 (b), BC-2 (d) and BJAB (f) cells from 3 independent experiments, and error bars represent the standard deviations. Statistically significant differences (*p ≤ 0.05, **p ≤ 0.01, paired 2-tailed t-test) between control and Abe-treated cells are indicated.

**Additional file 5. **Abe increases cell surface expression of ICAM-1, B7-2, and PD-L1 in PEL cell lines and EBV-infected BL cell lines. JSC-1 (a), BCBL-1 (b), Akata (c), and Raji (d) cells were treated with abemaciclib, or with RPMI medium control for 3 days. Surface expression of ICAM-1, B7-2 and PD-L1 were analyzed by flow cytometry. Results shown are one representative experiment of 3 separate experiments.

**Additional file 6. **CDK4/6 inhibitors increase cell surface expression of B7-2 in HUVEC and KSHV-infected HUVEC. HUVEC were either infected or mock infected with KSHV.BAC16, followed by culturing in the absence or presence of indicated concentrations of Abe, Pal, and Rib for 4 days. Results shown are histogram figures from 3 separate experiments.

**Additional file 7. **Abe enhances secretion of IL28B/ IL29 from PEL cells. JSC-1 and BCBL-1 cells were treated with 1 μM Abe, or with RPMI medium control for 3 days. The supernatant was then collected, purified, and analyzed by ELISA for IL28/IL29 production. The figure shows the average amount of secreted IL28B and IL29 from 3 independent experiments. Statistically significant differences (*p ≤ 0.05, paired 2-tailed t-test) between control and Abe-treated cells are indicated.

**Additional file 8. **Primers used for RT-qPCR.

## Data Availability

The materials and data used during the current study are available from the corresponding author on reasonable request.

## Abbreviations

Abe: Abemaciclib
BL: Burkitt’s lymphoma
DNMT1: DNA methyltransferase 1
EBV: Epstein–Barr virus
ERV: Endogenous retrovirus
FDA: Food and Drug Administration
GaCa: Gastric carcinomas
GFP: Green fluorescent protein
HL: Hodgkin's lymphoma
HUVEC: Human umbilical vein endothelial cells
ICAM-1: Intracellular adhesion molecule 1
KICS: KSHV inflammatory cytokine syndrome
KS: Kaposi sarcoma
KSHV: Kaposi sarcoma-associated herpesvirus
LANA: Latency-associated nuclear antigen
LMP-1: Latent membrane protein-1
MCD: Multicentric Castleman’s disease
MHC-I: Major histocompatibility antigen class I
NK: Natural killer
NPC: Undifferentiated nasopharyngeal carcinoma
Pal: Palbociclib
PEL: Primary effusion lymphoma
PT-LPD: Post-transplant lymphoproliferative disease
Rib: Ribociclib
vIL-10: Viral interleukin-10
vIL-6: Viral interleukin-6

## Declarations

This research was presented in part as an abstracts and poster at the 23rd International Workshop on Kaposi's Sarcoma Herpesvirus & Related Agents, Virtual meeting, (2021) and the 17th International Conference on Malignancies in HIV/AIDS, NIH, Bethesda, MD (2019).
