# Supplementary figures and images for: CDK4/6 inhibitors sensitize gammaherpesvirus-infected tumor cells to T-cell killing by enhancing expression of immune surface molecules
Source: J Transl Med. 2022 May 13;20:217. doi: 10.1186/s12967-022-03400-z (PMC9101822; doi:10.1186/s12967-022-03400-z)

**a****BC-1**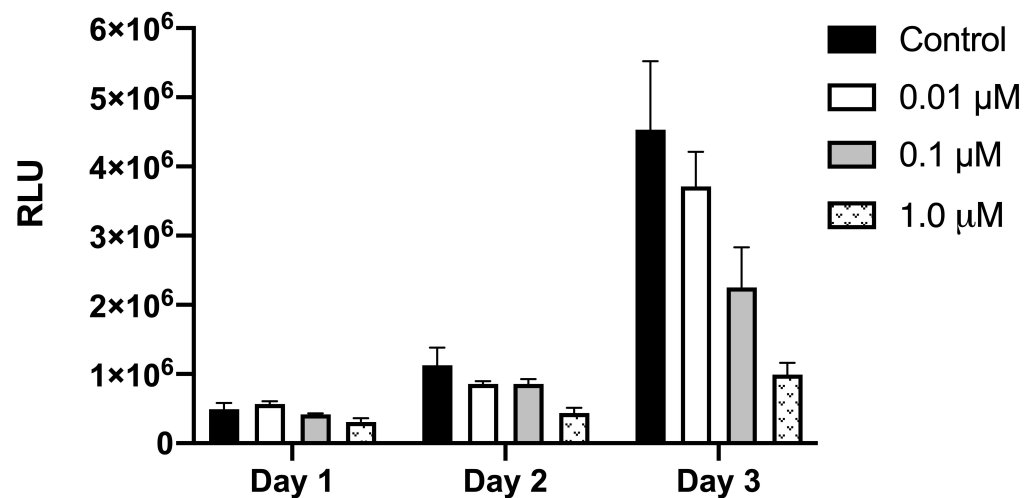**b****BC-2**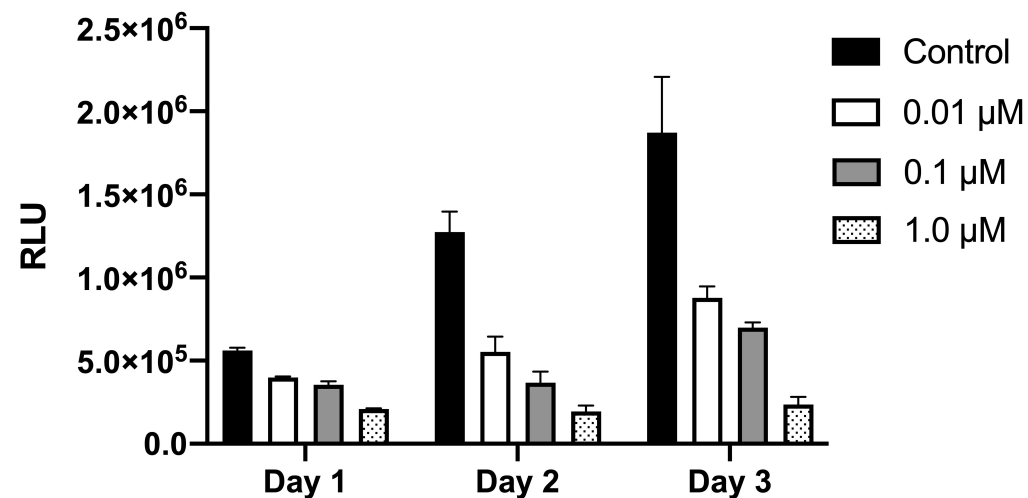**c****Daudi**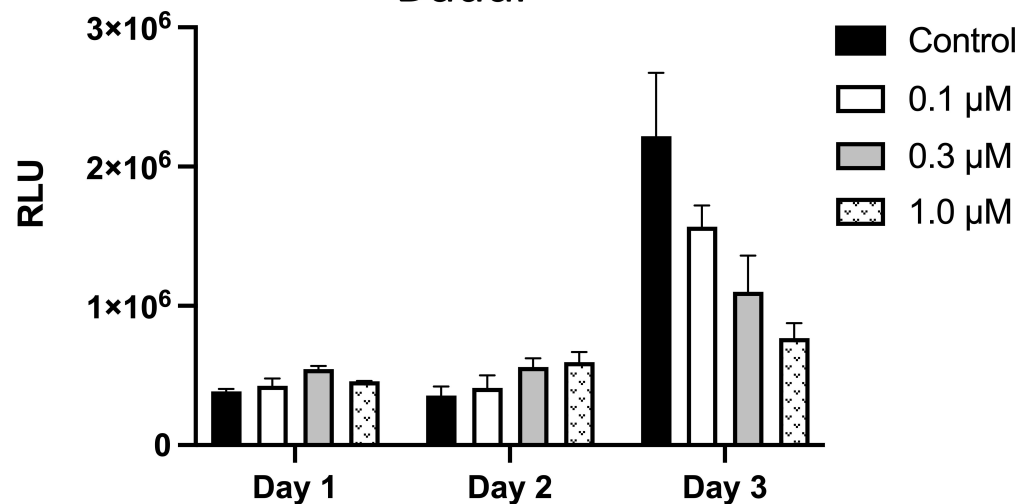**d****BJAB**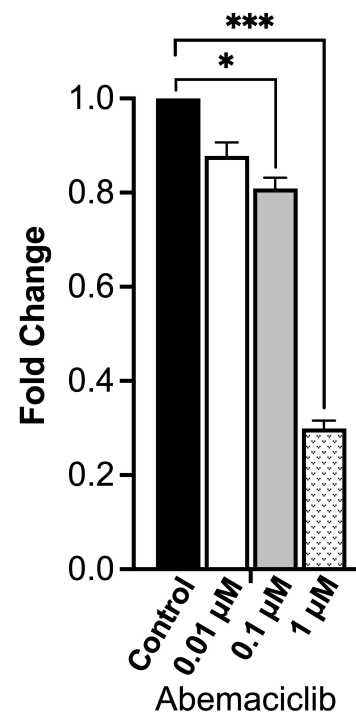**e****CA46**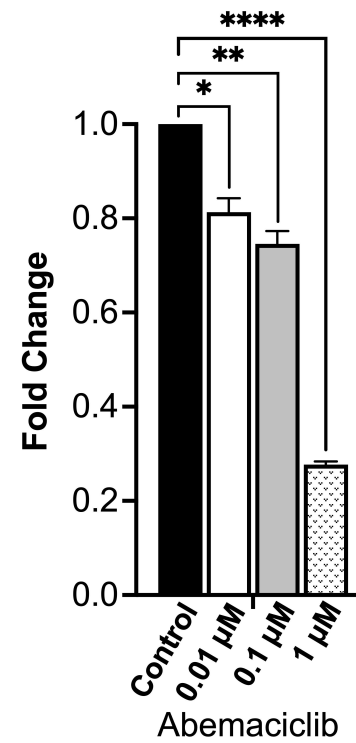

Supplement: Supplementary file 1 — Additional file 1. CDK4/6 inhibitors inhibit growth of a variety of PEL and BL cell lines, BC-1 (a), BC-2 (b), Daudi (c), BJAB (d), and CA46 (e) were treated in triplicate with indicated concentrations of abemaciclib, or with RPMI medium control for 1, 2, and 3 days (except BJAB and CA46 cells that were treated only for 3 days). The number of viable cells was assessed using CellTiter-Glo® Luminescent Cell Viability Assay. Shown is the data from one representative experiment. Error bars indicate the standard deviations from 3 technical replicates. [file 12967_2022_3400_MOESM1_ESM.pdf]

Brightfield

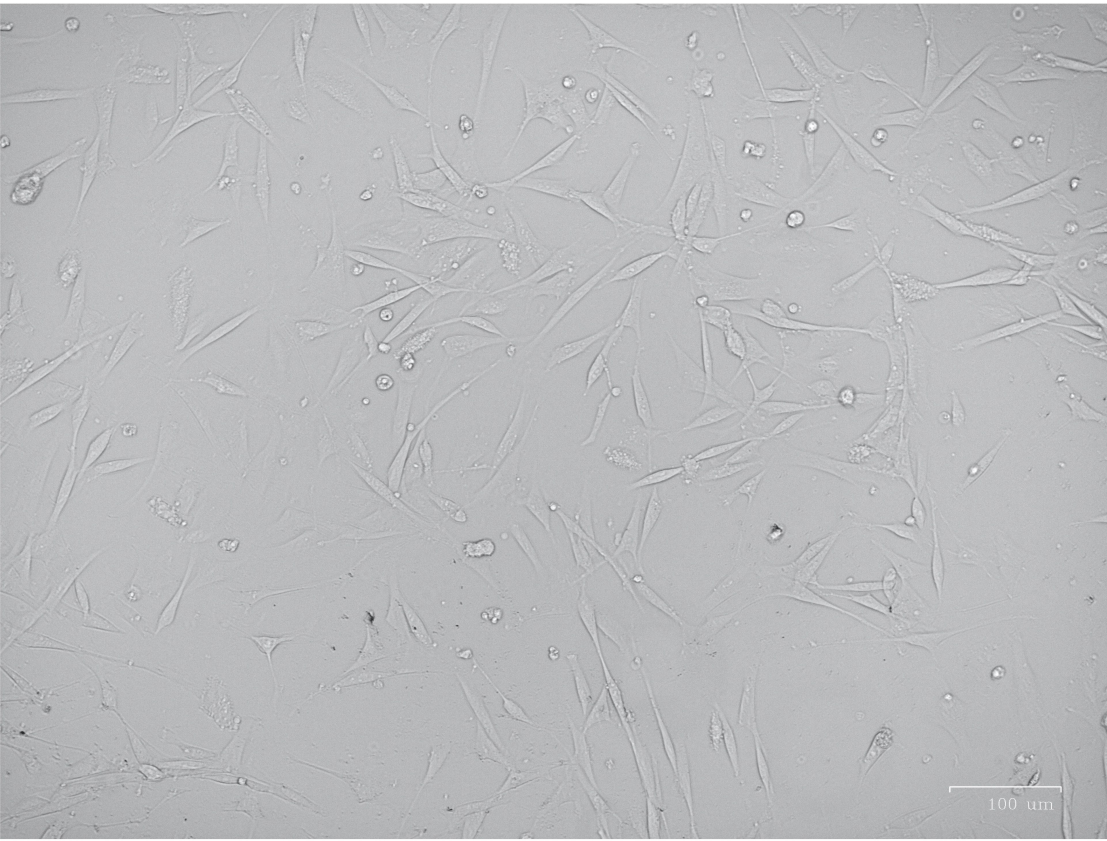

GFP

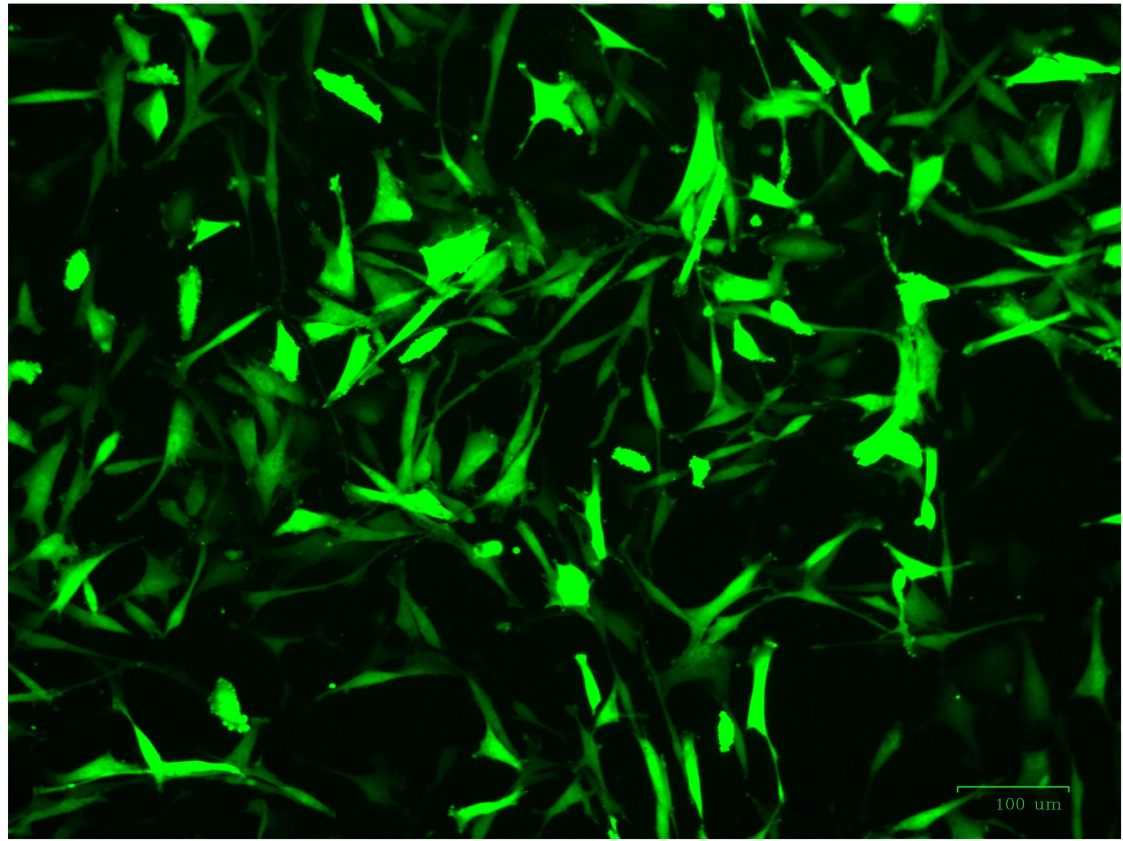

Supplement: Supplementary file 2 — Additional file 2. GFP expression in HUVEC infected by KSHV.BAC16. Cells were infected with diluted virus at a multiplicity of infection (MOI) of 15, as determined by LANA copy number, with 8 μg/ml polybrene. Virus supernatants were washed off after 8 h and replaced with fresh medium. The GFP signal was captured using the ZOE Fluorescent Cell Imager at 24 h post-infection. Shown are the brightfield view on the left and the GFP signal of the same field on the right. [file 12967_2022_3400_MOESM2_ESM.pdf]

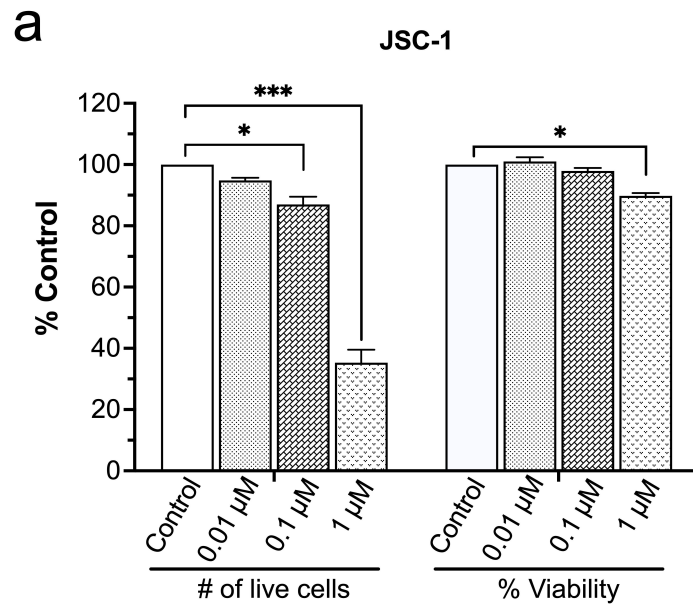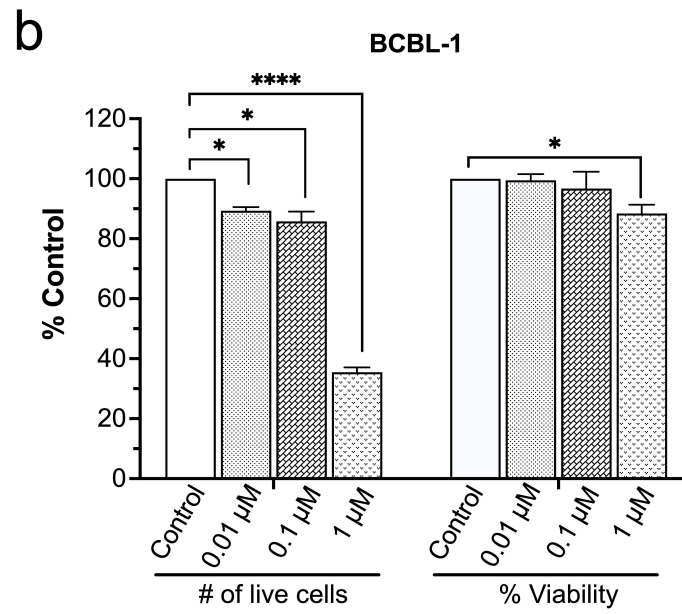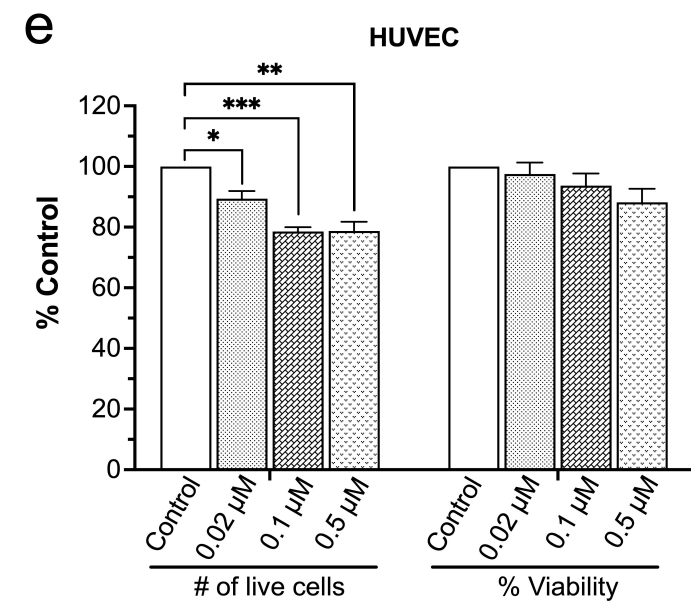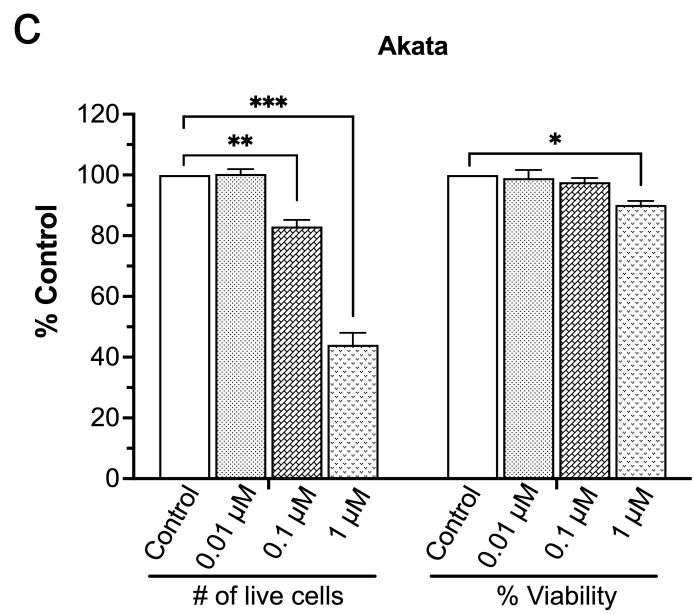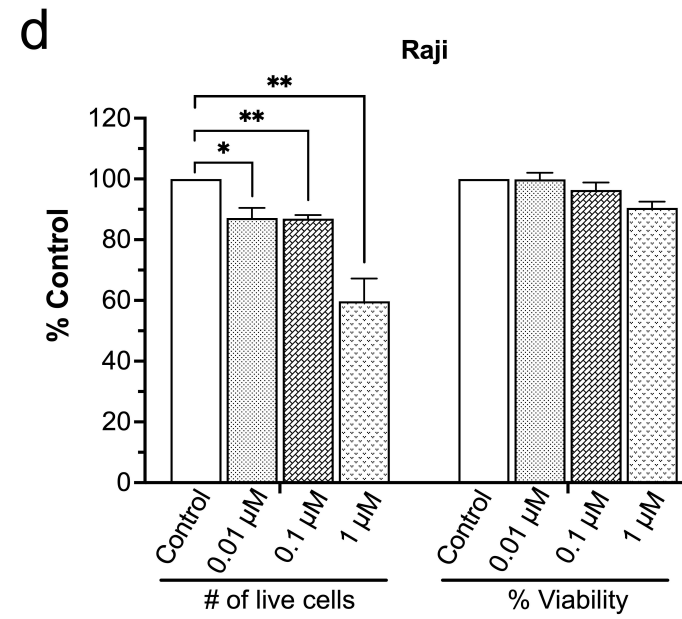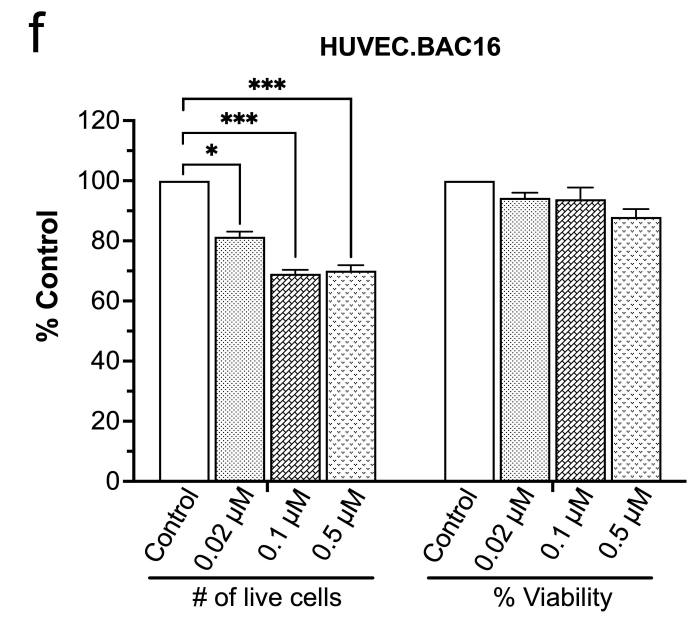

Supplement: Supplementary file 3 — Additional file 3. Abe reduces cell numbers but not the viability of KSHV+ cells and EBV+ cells. Cells were cultured in the absence or presence of indicated concentrations of Abe. After 3 days (JSC-1, BCBL-1, Akata and Raji) or 4 days (HUVEC and HUVEC.BAC.16), the number of viable cells was assessed using CellTiter-Glo® Luminescent Cell Viability Assay and the percentage of alive vs dead cells was assessed using trypan blue staining. Number of live cells and % viability (percentage of cells that are alive) for JSC-1 (a), BCBL-1 (b), Akata (c), Raji (d), HUVEC (e) and HUVEC.BAC16 (f) were calculated and presented as % of control-treated cells. Shown are the means from 3 separate experiments. Error bars indicate the standard deviations. Statistics were done using unpaired two-tailed t-test. Asterisks indicate p values: *p < 0.05, **p < 0.01, ***p < 0.001. Those without asterisks are all not significant (p ≥ 0.05). [file 12967_2022_3400_MOESM3_ESM.pdf]

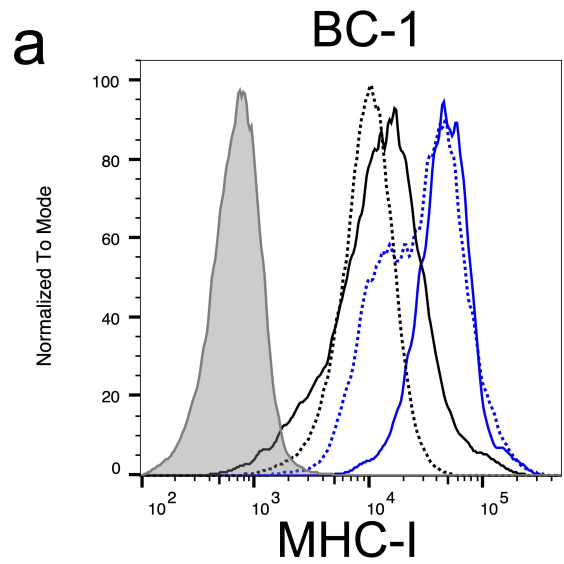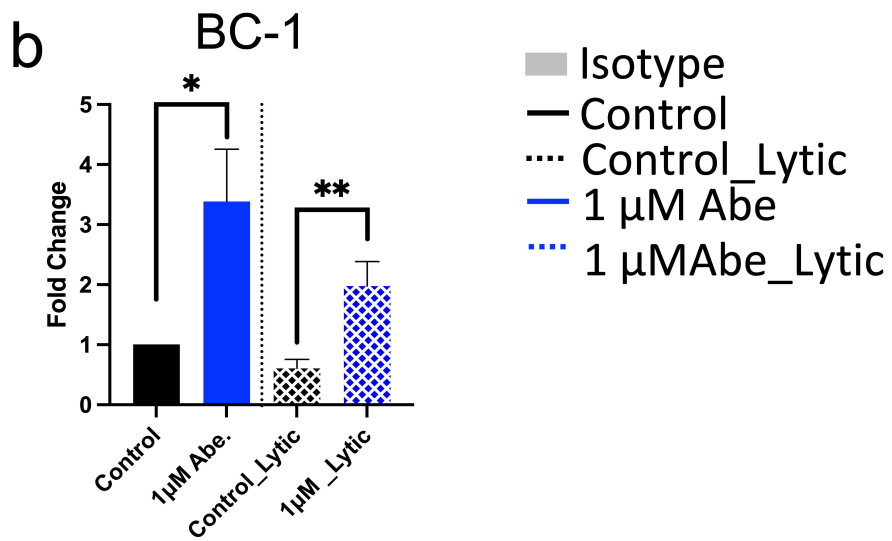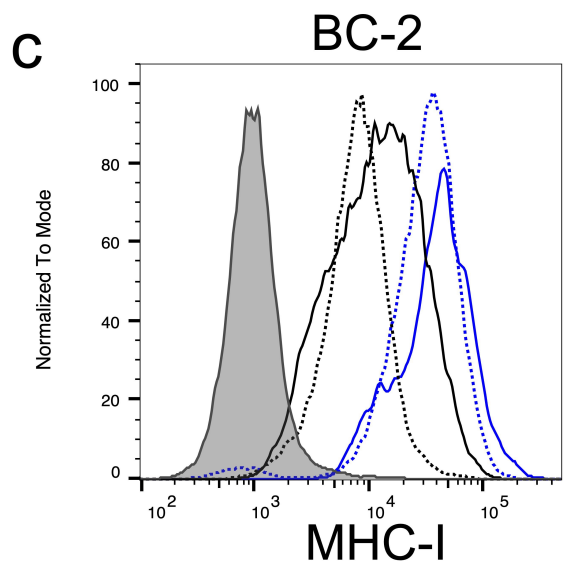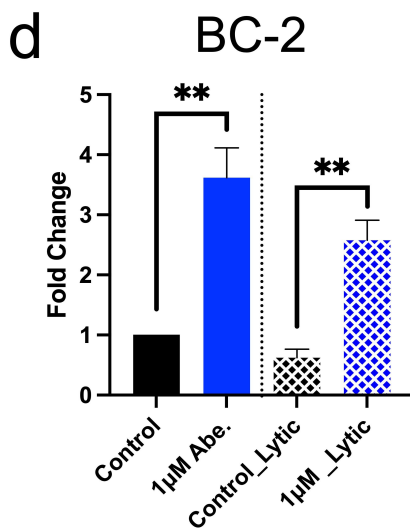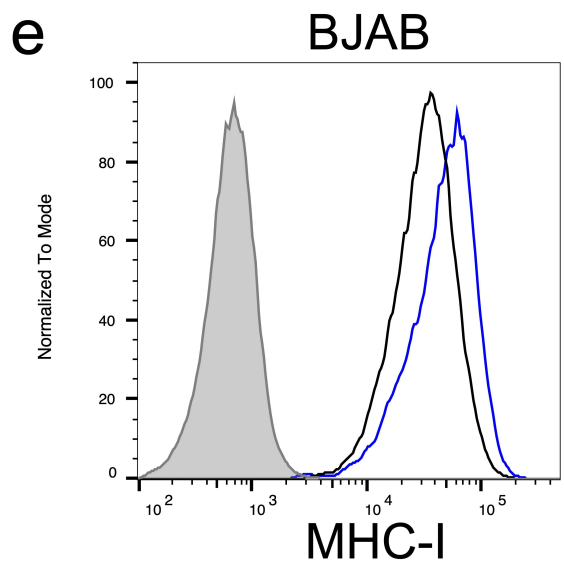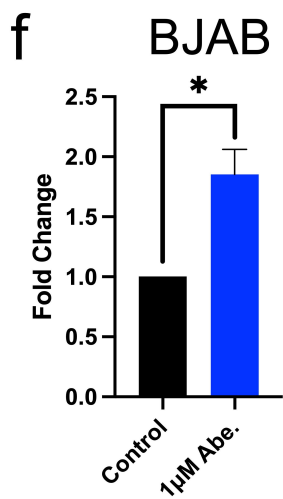

Supplement: Supplementary file 4 — Additional file 4. Effects of Abe on MHC-I surface expression in BC-1 and BC-2 PEL lines and BJAB, an EBV-uninfected BL cell lines. Cells were treated either for 48 h (BC-1 (a and b) and BC-2 (c and d)) or 72 h (BJAB (e and f)), with 1 μM Abe or RPMI medium control. PEL cells (a-d) were then treated with sodium butyrate (0.3 mM) for another 24 h. All the cells were then analyzed by flow cytometry for surface MHC-I expression. Figures a, c, and e show a representative experiment. Figures b, d, and f show the mean fold change of MHC-I expression in BC-1 (b), BC-2 (d) and BJAB (f) cells from 3 independent experiments, and error bars represent the standard deviations. Statistically significant differences (*p ≤ 0.05, **p ≤ 0.01, paired 2-tailed t-test) between control and Abe-treated cells are indicated. [file 12967_2022_3400_MOESM4_ESM.pdf]

**a**

JSC-1

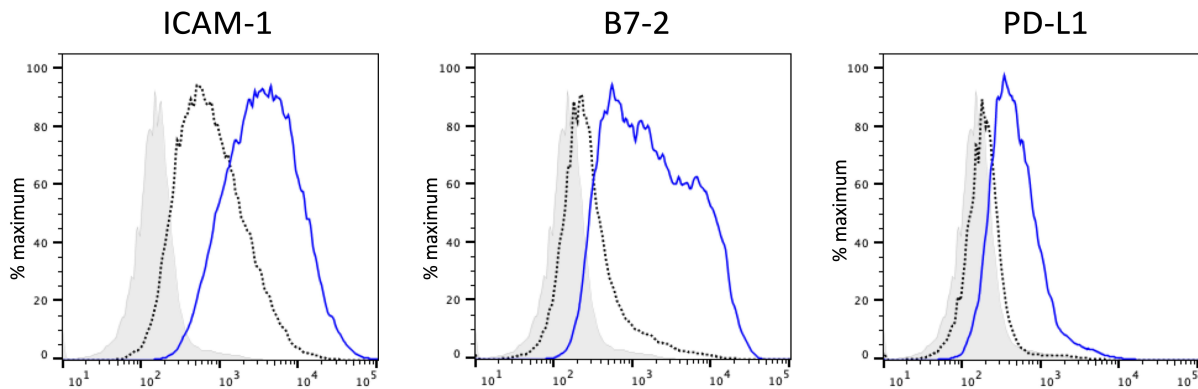**b**

BCBL-1

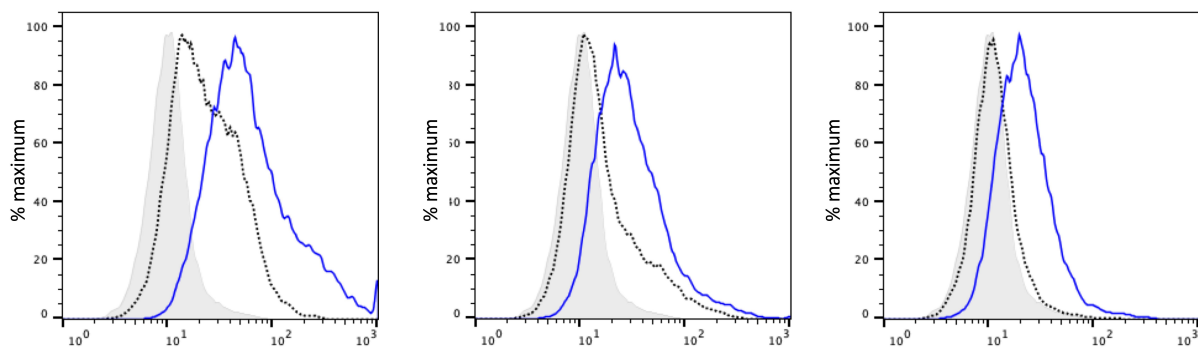**c**

Akata

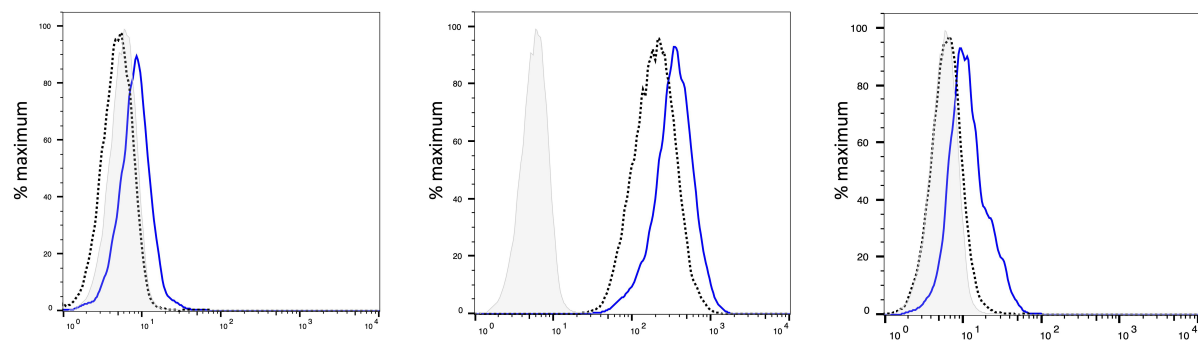**d**

Raji

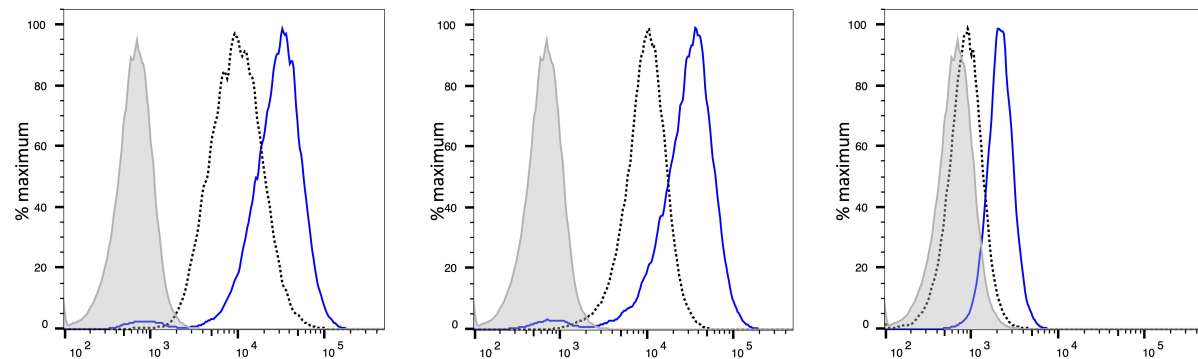

Supplement: Supplementary file 5 — Additional file 5. Abe increases cell surface expression of ICAM-1, B7-2, and PD-L1 in PEL cell lines and EBV-infected BL cell lines. JSC-1 (a), BCBL-1 (b), Akata (c), and Raji (d) cells were treated with abemaciclib, or with RPMI medium control for 3 days. Surface expression of ICAM-1, B7-2 and PD-L1 were analyzed by flow cytometry. Results shown are one representative experiment of 3 separate experiments. [file 12967_2022_3400_MOESM5_ESM.pdf]

# IL28B and IL29

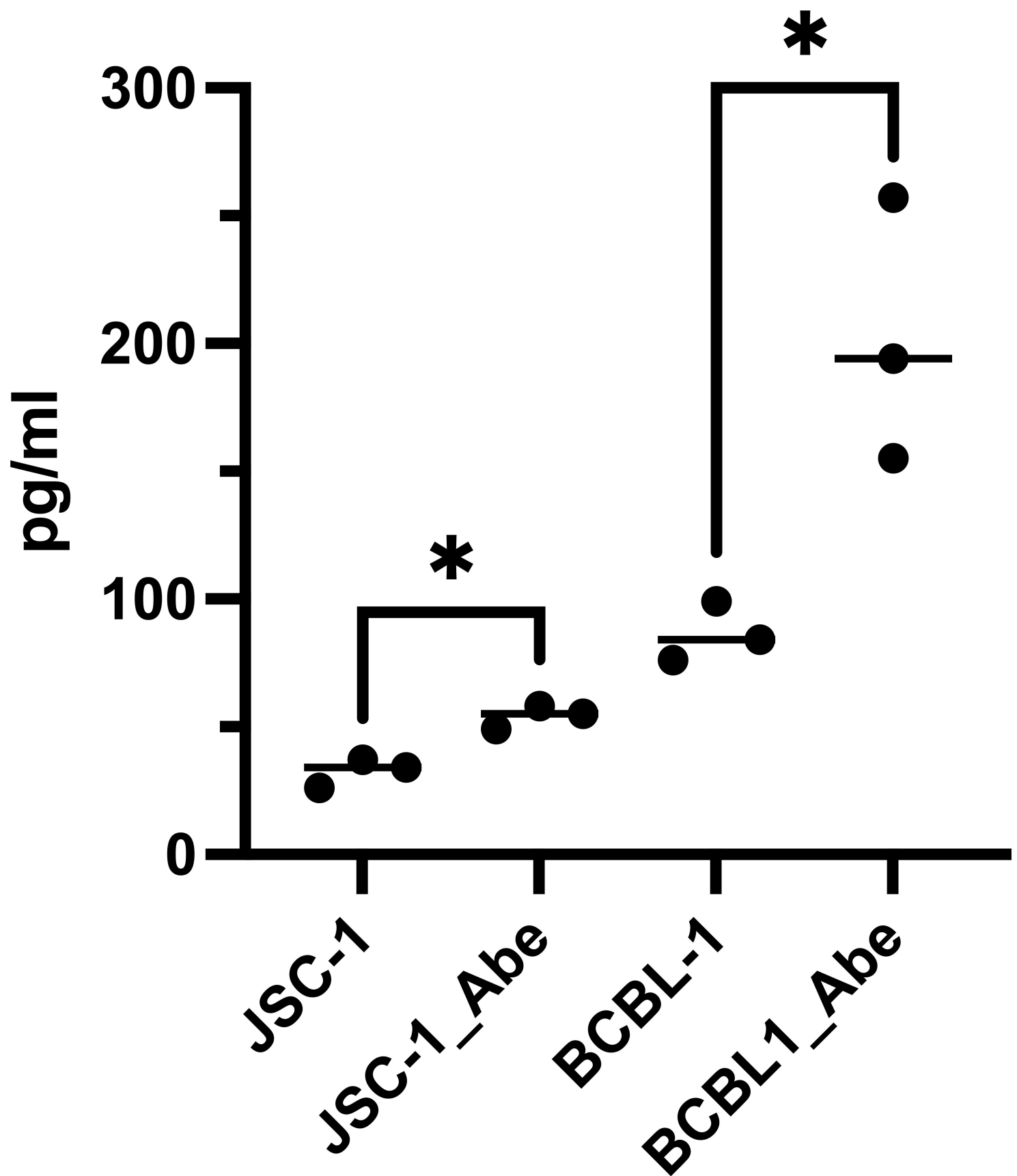

Supplement: Supplementary file 7 — Additional file 7. Abe enhances secretion of IL28B/ IL29 from PEL cells. JSC-1 and BCBL-1 cells were treated with 1 μM Abe, or with RPMI medium control for 3 days. The supernatant was then collected, purified, and analyzed by ELISA for IL28/IL29 production. The figure shows the average amount of secreted IL28B and IL29 from 3 independent experiments. Statistically significant differences (*p ≤ 0.05, paired 2-tailed t-test) between control and Abe-treated cells are indicated. [file 12967_2022_3400_MOESM7_ESM.pdf]
