# Supplementary material for: CDK4/6 inhibitors sensitize gammaherpesvirus-infected tumor cells to T-cell killing by enhancing expression of immune surface molecules
Source: J Transl Med. 2022 May 13;20:217. doi: 10.1186/s12967-022-03400-z (PMC9101822; doi:10.1186/s12967-022-03400-z)
Supplement: Supplementary file 6 — Additional file 6. CDK4/6 inhibitors increase cell surface expression of B7-2 in HUVEC and KSHV-infected HUVEC. HUVEC were either infected or mock infected with KSHV.BAC16, followed by culturing in the absence or presence of indicated concentrations of Abe, Pal, and Rib for 4 days. Results shown are histogram figures from 3 separate experiments. [file 12967_2022_3400_MOESM6_ESM.pdf]

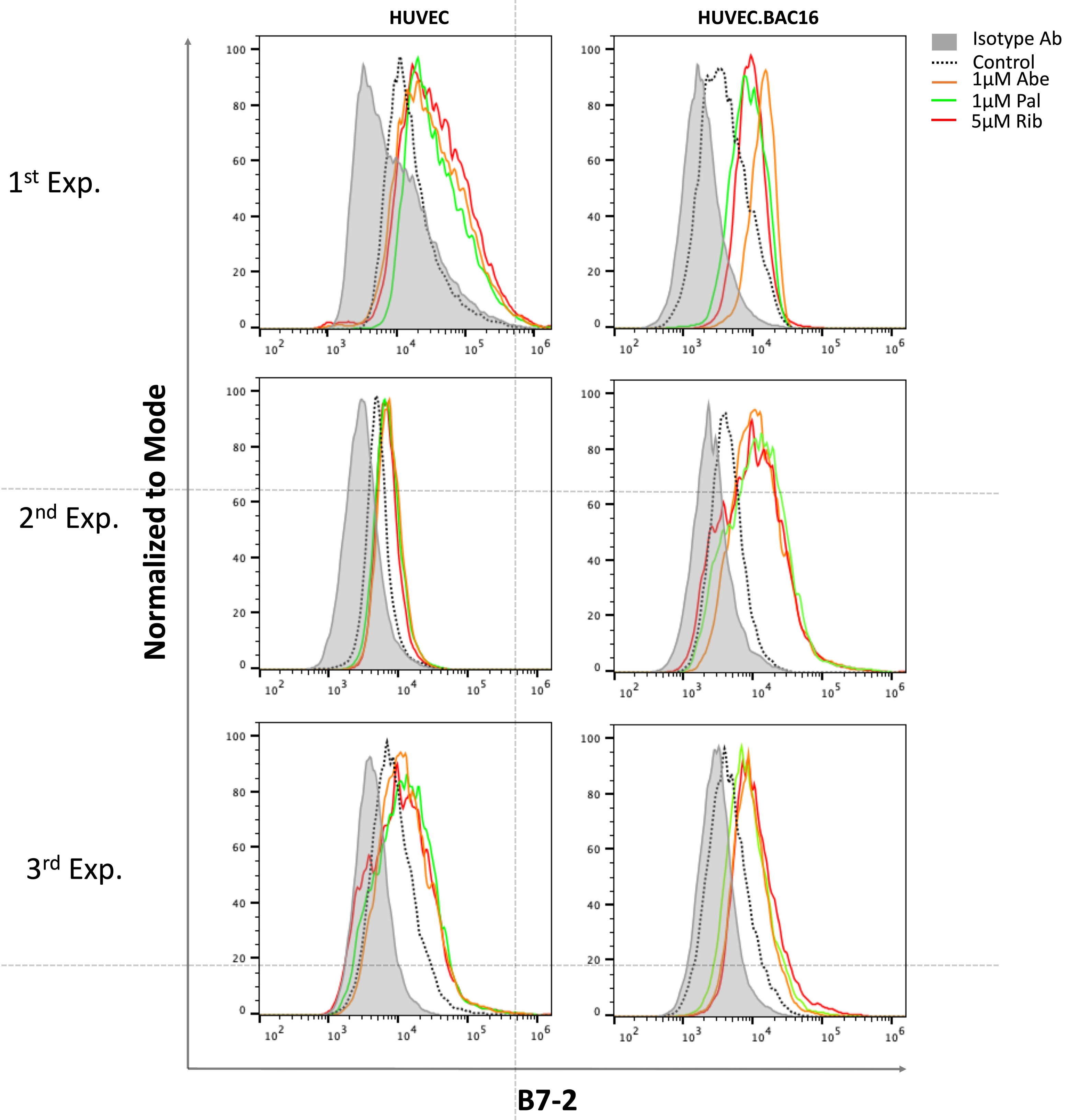

HUVEC
HUVEC.BAC16
Isotype Ab
Control
1μM Abe
1μM Pal
5μM Rib
1st Exp.
2nd Exp.
3rd Exp.
Normalized to Mode
B7-2
