# Supplementary material for: CDK4/6 inhibitors sensitize gammaherpesvirus-infected tumor cells to T-cell killing by enhancing expression of immune surface molecules
Source: J Transl Med. 2022 May 13;20:217. doi: 10.1186/s12967-022-03400-z (PMC9101822; doi:10.1186/s12967-022-03400-z)
Supplement: Supplementary file 8 — Additional file 8. Primers used for RT-qPCR. [file 12967_2022_3400_MOESM8_ESM.pdf]

Primers used for RT-qPCR

Gene Name	Forward Primer Sequence (5' to 3')	Reverse Primer Sequence (5' to 3')
ORF73 (LANA)	CGCGAATACCGCTATGTACTCA	GGAACGCGCCTCATACGA
ORF50 (RTA)	TTGCCAAGTTTGTACAACTGCT	ACCTTGCAAAGACCATTCAGAT
K2 (vIL6)	CTGTTACCGTACCGGCATCT	GGGTGGACTGTAGTGCGTCT
ORF45	TCGTGAACCAATCCCAGCCAAG	TCCACCCAGTTTGAGAAGCATC
ORF57	CATCCTAGAGGACTCTGT	TTGCTCGTCTTCCAGTGT
EBER2	GGACAGCCGTTGCCCTAGTGG	AGCGGACAAGCCGAATACCCTTC
BMRF1	CGTGCCAATCTTGAGGTTTT	CGGAGGCGTGGTTAAATAAA
ACTB	CCTTCCTGGGCATGGAGT	CAGGGCAGTGATCTCCTTCT
HLA	GAGATCACACTGACCTGGCA	GAACCTTCCAGAAGTGGG
CD54	AGCGGCTGACGTGTGCAGTAAT	TCTGAGACCTCTGGCTTCGTCA
CD86	CCATCAGCTTGTCTGTTTCATTCC	GCTGTAATCCAAGGAATGTGGTC
CD274	TGCCGACTACAAGCGAATTACTG	CTGCTTGTCCAGATGACTTCGG
DDX58	TGTGCTCCTACAGGTTGTGGA	CACTGGGATCTGATTCGCAAAA
DNMT1	CCTAGCCCCAGGATTACAAGG	ACTCATCCGATTTGGCTCTTTC
ERV3-1	TACGTGGTCGGGGAACATCAT	GGTCACAGACTGAGTAGGTTGT
IFIT1	GCGCTGGGTATGCGATCTC	CAGCCTGCCTTAGGGGAAG
IFNL2	ACGCGAGACCTGAATTGTGT	AGCGACTGGGTGGCAATAAA
NLRC5	GCTCGGCAACAAGAACCTGT	GGTCCAAGGTCTCGTTCCT
OAS2	GGAGCTTCCTGATTGGCAGA	ATGTAGGGTGGCAAGCACTG
STAT1	CAGCTTGACTCAAAATTCCTGGA	TGAAGATTACGCTTGCTTTTCCT
IFN-α	GACTCCATCTTGGCTGTGA	TGATTTCTGCTCTGACAACCT
IFN-β	CTTGGATTCCTACAAAGAAGCAGC	TCCTCCTTCTGGAACTGCTGCA
IFN-γ	GAGTGTGGAGACCATCAAGGAAG	TGCTTTGCGTTGGACATTCAAGTC
